# Efferocytosis reprograms the tumor microenvironment to promote pancreatic cancer liver metastasis

**DOI:** 10.1038/s43018-024-00731-2

**Published:** 2024-02-14

**Authors:** Yuliana Astuti, Meirion Raymant, Valeria Quaranta, Kim Clarke, Maidinaimu Abudula, Olivia Smith, Gaia Bellomo, Vatshala Chandran-Gorner, Craig Nourse, Christopher Halloran, Paula Ghaneh, Daniel Palmer, Robert P. Jones, Fiona Campbell, Jeffrey W. Pollard, Jennifer P. Morton, Ainhoa Mielgo, Michael C. Schmid

**Affiliations:** 1 Department of Molecular and Clinical Cancer Medicine, University of Liverpool, Liverpool, UK; 2 Computational Biology Facility, University of Liverpool, Liverpool, UK; 3 Cancer Research UK Scotland Institute, Glasgow, UK; 4 School of Cancer Sciences, University of Glasgow, Glasgow, UK

**Keywords:** Cancer microenvironment, Metastasis, Tumour immunology, Cancer

## Abstract

Pancreatic ductal adenocarcinoma is a highly metastatic disease and macrophages support liver metastases. Efferocytosis, or engulfment of apoptotic cells by macrophages, is an essential process in tissue homeostasis and wound healing, but its role in metastasis is less well understood. Here, we found that the colonization of the hepatic metastatic site is accompanied by low-grade tissue injury and that efferocytosis-mediated clearance of parenchymal dead cells promotes macrophage reprogramming and liver metastasis. Mechanistically, progranulin expression in macrophages is necessary for efficient efferocytosis by controlling lysosomal acidification via cystic fibrosis transmembrane conductance regulator and the degradation of lysosomal cargo, resulting in LXRα/RXRα-mediated macrophage conversion and upregulation of arginase 1. Pharmacological blockade of efferocytosis or macrophage-specific genetic depletion of progranulin impairs macrophage conversion, improves CD8^+^ T cell functions, and reduces liver metastasis. Our findings reveal how hard-wired functions of macrophages in tissue repair contribute to liver metastasis and identify potential targets for prevention of pancreatic ductal adenocarcinoma liver metastasis.

## Abstract (web summary)

Schmid and colleagues show that pancreatic cancer cell colonization of the liver is accompanied by low-grade tissue injury and efferocytosis, which promotes reprogramming of macrophages and upregulation of arginase.

## Main

Pancreatic ductal adenocarcinoma (PDAC) is a highly metastatic disease with a 5-year survival rate of less than 7%^1^. Metastatic spread commonly occurs to the liver and is the primary cause of death for patients with PDAC^2^. By the time that PDAC is diagnosed, the majority of patients (~60%) have non-resectable metastatic cancer^3^. Around 70% of the patients whose primary tumor is removed relapse with hepatic metastasis within 2 years of surgery^4^. Understanding the mechanisms underlying metastasis in pancreatic cancer is critical to improve outcomes for these patients.

Clearance of dying cells is fundamental to homeostasis, tissue repair and disease^5^. Efferocytosis, the engulfment of dead cells by phagocytes, is performed predominantly by macrophages^6^. Macrophages are critical immune cells and are highly plastic, acquiring tumor repressive or supportive functions depending on the context^7,8^. PDAC liver metastasis is accompanied by macrophage accumulation^9,10^, with macrophages often displaying an immunosuppressive phenotype, promoting tumor growth and limiting the impact of immunotherapy^11–13^.

Emerging studies indicate that tumor-associated macrophages can coexist within the same tumor as immunostimulatory and immunosuppressive subtypes^14^. Therapeutically targeting immunosuppressive macrophages holds great promise to improve current treatment options for patients with cancer. In this study, using single-cell analysis, we identified an efferocytic macrophage population in the early stage of PDAC liver metastasis that displays potent immunosuppressive activity. We found that efferocytosis reprograms macrophages toward an immunosuppressive phenotype and that genetic or pharmacological inhibition of efferocytic macrophages restored tumor immunity in PDAC liver metastasis and impaired metastatic outgrowth.

## Results

### Macrophage populations in metastatic livers are diverse

**Fig. 1:**
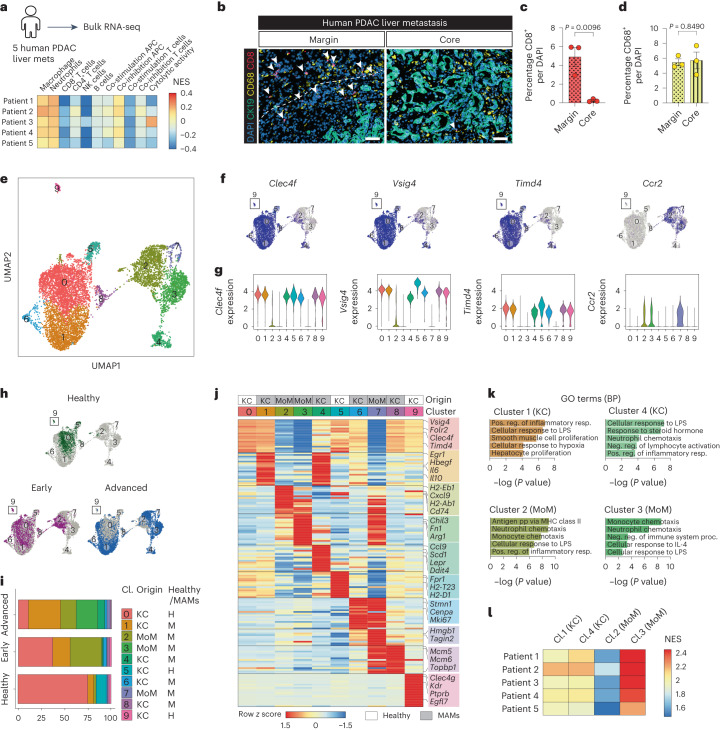
Identification of MAM populations in metastatic PDAC livers by single-cell RNA sequencing combined with spatial in situ labeling. **a**, Schematic of bulk RNA sequencing on fresh liver metastasis biopsies from chemotherapy-naive patients with PDAC (*n* = 5) (top) and heat map showing scores (normalized enrichment score (NES); single-sample gene set enrichment analysis (ssGSEA)) for immune signatures (bottom). NK, natural killer. **b**–**d**, Representative immunofluorescence images (**b**) and quantification of CD8^+^ T cells (**c**) and macrophages (CD68^+^) (**d**) in the tumor margin and core of human PDAC liver metastasis (*n* = 3 patients). Cancer cells were indicated by CK19^+^ staining. Arrowheads indicate CD8^+^ T cells. Scale bars, 50 µm. Error bars, mean ± s.e.m. *P* values, two-tailed unpaired *t*-test. DAPI, 4,6-diamidino-2-phenylindole. **e**, Uniform Manifold Approximation and Projection (UMAP) plot identifying ten clusters within macrophages (F4/80^+^) isolated by flow cytometry from healthy liver, early metastatic livers (d5) and advanced metastatic livers (d10) induced by intra-portal implantation of KPC-derived cells into mice with established orthotopic PDAC tumors (*n* = 3 mice per group). **f**,**g**, UMAP plots (**f**) and violin plots (**g**) depicting expression of common markers of KCs (*Clec4f*, *Vsig4* and *Timd4*) and MoMs (*Ccr2*) in the scRNA-seq dataset. **h**,**i**, UMAP plots (**h**) and bar chart (**i**) depicting distribution of different macrophage clusters in healthy livers, early metastatic livers (d5) and advanced metastatic livers (d10). **j**, Heat map depicting relative average expression of the top upregulated differentially expressed genes in each macrophage cluster compared to all other clusters in the scRNA-seq dataset. Representative genes are labeled for each cluster. **k**, Enriched Gene Ontology (GO) biological processes (BP) in major MAM clusters derived from KCs (cluster 1 and 4) and monocytes (cluster 2 and 3). Statistical enrichment analyses were performed using Fisher’s exact test on g:Profiler. LPS, lipopolysaccharide; pp, processing and presentation; MHC, major histocompatibility complex; IL, interleukin. **l**, Heat map showing signature scores (NES; ssGSEA) of major KC-MAM and Mo-MAM clusters in human PDAC liver metastasis samples (*n* = 5 patients). Source data

**Extended Data Fig. 1:**
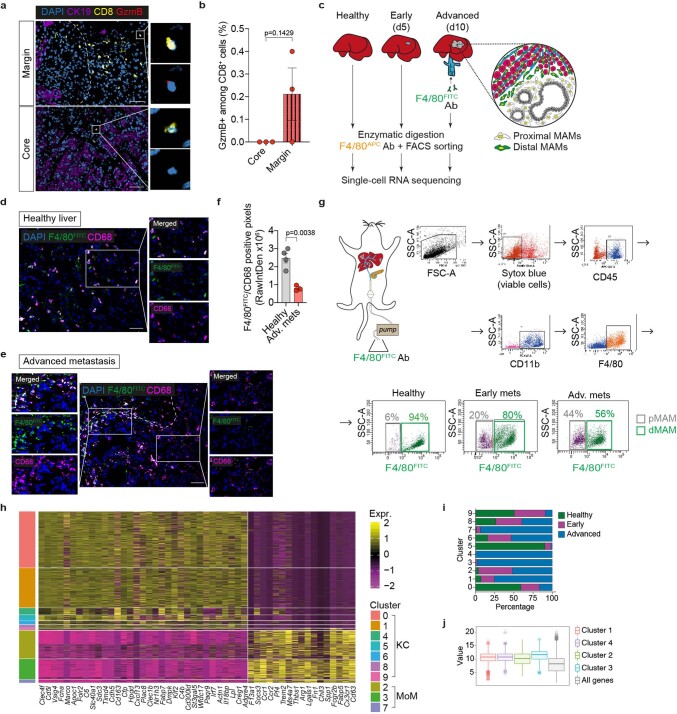
Single cell analysis with spatial in-situ labeling identifies heterogeneity in metastasis-associated macrophages. **(a-b)** Representative immunofluorescence images **(a)** and quantification **(b)** of cytotoxic GzmB^+^ CD8^+^ T cells in human PDAC liver metastasis (n = 3 patients). Cancer cells were indicated by CK19^+^ staining. Scale bars, 50 µm. Error bars, mean ± SEM. *P*-value, two-tailed unpaired t-test. **(c)** Schematic of the scRNA sequencing approach combined with a spatial in-situ labeling strategy. MAMs=metastasis associated macrophages. **(d,e)** Representative immunofluorescence images of healthy livers **(d)** and advanced stage (d10) **(e)** of liver metastasis following *in situ* labeling with FITC-conjugated F4/80 antibody (n = 3 mice/group, from one experiment) via retrograde perfusion, followed by *ex vivo* staining with pan-macrophage marker CD68. Scale bars, 50 µm. **(f)** Quantification of double-labeled macrophages (F4/80^FITC+^/CD68^+^) in healthy and tumor bearing livers (healthy n = 4 mice, metastasis n = 3 mice) as shown in **(d, e)**. Error bars, mean ± SEM. *P*-value, two-tailed unpaired t-test. **(g)** FACS gating strategy for sorting of proximal (pMAMs; F4/80^APC+^F4/80^FITC−^) and distal metastasis-associated macrophages (dMAMs; F4/80^APC+^F4/80^FITC+^). **(h)** Heatmap depicting expressions of the most enriched genes in KC vs MoM clusters in the scRNAseq dataset. **(i)** Proportions of each macrophage cluster identified in the scRNAseq in healthy liver, early metastasis, and advanced metastasis. **(j)** Expression values of the genes in the cluster 1-4 signatures from scRNAseq analyses versus all genes in the human samples (cluster 1 n = 751 genes; cluster 4 n = 291 genes; cluster 2 n = 255 genes; cluster 3 n = 310 genes). Boxplots indicate the median, first and third quartiles (hinges), and outlier points extend beyond 1.5× interquartile ranges from either hinge (whiskers). Source data

To investigate the immunological status of advanced metastatic lesions in PDAC, we collected fresh liver biopsies from treatment-naive metastatic patients with PDAC and performed bulk RNA sequencing. We found that metastatic lesions are immune silenced, enriched in macrophages and neutrophils, with low signature scores for T cells, B cells and natural killer cells (Fig. 1a). Immunofluorescence tissue staining of advanced metastatic PDAC lesions showed that the lesion periphery is rich in macrophages (CD68^+^) and mostly granzyme B-negative T cells (CD8^+^) (Fig. 1b–d and Extended Data Fig. 1a,b). Macrophages were also abundant in the metastatic core; however, CD8^+^ T cells were virtually absent (Fig. 1b–d). Thus, the metastatic tumor microenvironment in PDAC is immunosuppressive, with high numbers of metastasis-associated macrophages (MAMs) and poor CD8^+^ T cell infiltration.

To characterize MAM heterogeneity in pancreatic cancer liver metastasis in vivo, we induced liver metastasis by intra-portal injection of cells isolated from the genetically engineered ‘KPC’ mouse model of PDAC (*Kras*^G12D^; *Trp53*^R172H^; *Pdx1-Cre*), in orthotopic PDAC tumor-bearing mice. MAMs were isolated by flow cytometry and analyzed using single-cell RNA sequencing (scRNA-seq) (Extended Data Fig. 1c). We resolved the spatial distribution of MAMs from early (day 5) and advanced metastatic lesions (day 10) using an in situ antibody-labeling approach. Established metastatic liver tumors in PDAC are highly fibrotic and poorly vascularized^15^. Perfusion with FITC-conjugated F4/80 (F4/80^FITC^) antibody solution therefore mainly stains macrophages in the peripheral area of metastatic lesions (F4/80^FITC+^); intra-metastatic macrophages remain unstained (F4/80^FITC–^) (Extended Data Fig. 1d–g). We define unstained macrophages as metastasis-proximal macrophages (pMAMs) and stained macrophages as metastasis-distal macrophages (dMAMs).

We analyzed macrophages from healthy livers (2,428 cells), early metastasis (2,007 cells), advanced proximal metastasis (1,953 cells) and advanced distal metastasis (2,783 cells). Ten distinct populations were revealed after Seurat-based clustering and dimensionality reduction (Fig. 1e). Tissue resident Kupffer cells (KCs) and monocyte-derived macrophages (MoMs) comprise the macrophage population in the liver^16^. Clusters 0, 1, 4, 5, 6, 8 and 9 showed high expression of KC markers such as *Clec4f, Vsig4*, and *Timd4* (ref. ^17^) (Fig. 1f,g and Extended Data Fig. 1h). In parallel, clusters 2, 3 and 7 expressed monocyte-derived macrophage (MoM) marker *Ccr2* (ref. ^18^) (Fig. 1f,g and Extended Data Fig. 1h).

In tumor-free livers KC subsets dominated the macrophage population, especially cluster 0 at 75% of the population (Fig. 1h,i), confirming previous reports^17^. In livers with metastatic lesions, KC clusters 1, 4, 6 and 8 and all three MoM clusters were expanded compared to tumor-free livers and were therefore identified as metastasis-induced macrophages (Fig. 1h,i and Extended Data Fig. 1i). Differentially expressed gene (DEG) signature analysis confirmed that cluster 0 showed high expression of KC genes^17^, including *Vsig4*, *C6*, *Folr2*, *Clec4f*, *Apoc1* and *Timd4* (Fig. 1j and Supplementary Table 1), which were also highly expressed in the KC clusters 1, 5, 6, 8 and 9. In addition to these pan-KC markers, cluster 5 (10% of naive liver macrophages) highly expressed antigen-processing and presentation (AP) genes (*H2-Eb1*, *H2-Ab1*, *H2-T23* and *H2-D1*) (Fig. 1j and Supplementary Table 1). Cluster 9 (2% of naive liver macrophages) showed endothelial-like characteristics by their high expressions of *Clec4g*, *Kdr*, *Ptprb* and *Egfl7* (Fig. 1j and Supplementary Table 1).

Two major KC-MAM clusters, clusters 1 and 4, showed an inflammatory signature with high expression of pro-inflammatory cytokines, such as *Il6*, *Il1a*, *Il1b* and *Il18*, and NF-κB and MAPK signaling pathway genes (*Nfkbiz*, *Fosb*, *Trim25*, *Nfkbia* and *Map3k8*) (Fig. 1j,k and Supplementary Table 1). Inflammation repressors *Egr1* and *Il10* were also expressed, suggesting a negative feedback mechanism (Fig. 1j,k and Supplementary Table 1). Cluster 4 was uniquely characterized by expression of the leptin receptor *Lepr*, plus high expression of *Scd1*, *Tnf*, *Il1b* and *Il6*, suggesting a pro-fibrogenic phenotype^19,20^. The remaining KC-MAM subsets showed high expression of cell-cycle genes (*Stmn1*, *Cenpa* and *Mki67*) in cluster 6 and DNA replication genes (*Mcm5*, *Mcm6* and *Topbp1*) in cluster 8, characteristic of proliferating macrophages (Fig. 1j and Supplementary Table 1).

Within monocyte-derived MAM subsets, cluster 2 exhibited AP gene signatures, demonstrated by high expression of *H2-Eb1*, *H2-Ab1* and *Cd74* (Fig. 1j,k and Supplementary Table 1). Conversely, cluster 3 showed enrichment of *Chil3*, *Mrc1* and *Arg1*, suggesting an M2-like/immunosuppressive phenotype (Fig. 1j,k and Supplementary Table 1). The minor MoM population, cluster 7, displayed similar expression of *Chil3* and *Arg1* and high expression of cell-cycle genes (*Stmn1*, *Cenpa* and *Mki67*), suggesting that it is a proliferating subset of cluster 3 (Fig. 1j and Supplementary Table 1).

We explored whether the identified MAM signatures were present in our human PDAC liver metastasis RNA-seq data. All four MAM signatures (KC clusters 1 and 4 and MoM clusters 2 and 3) were detectable in human data (Extended Data Fig. 1j). Our scRNA-seq analysis showed cluster 3 MoMs to be the most abundant subset in core areas of advanced metastasis. In agreement with this, we found the highest signature score for this cluster in all liver biopsies taken from cores of advanced metastatic tumors of patients with PDAC (Fig. 1l).

Together, these data suggest that PDAC metastasis increases macrophage heterogeneity within both MoM and KC subsets and that immunosuppressive and immunostimulatory macrophage subpopulations coexist.

### CD74^−/low^ MoMs display potent immunosuppressive functions

**Fig. 2:**
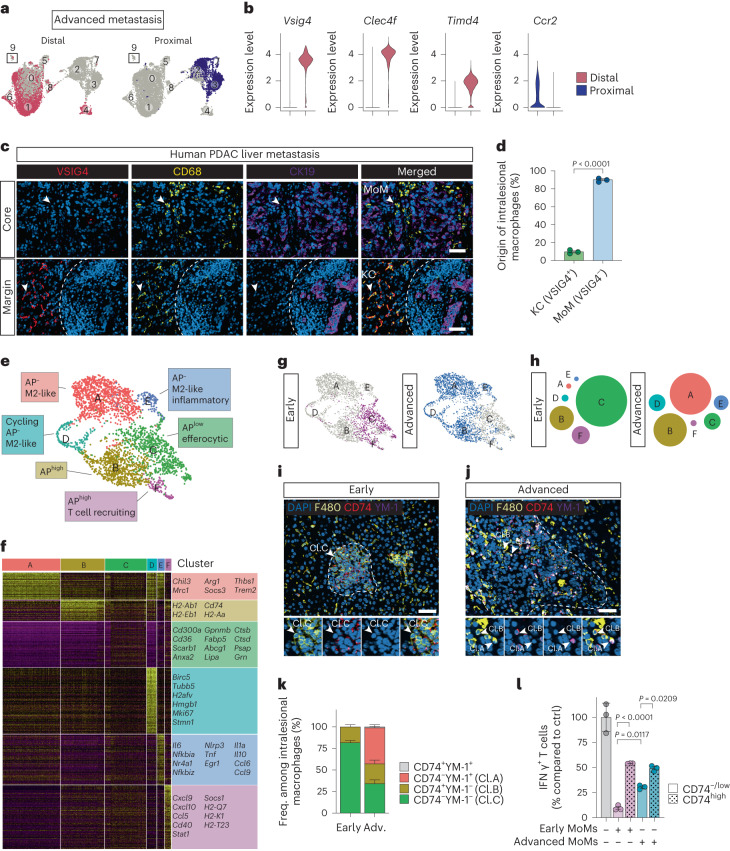
CD74^neg/low^ MoMs display potent immunosuppressive functions at an early metastatic stage. **a**, UMAP plots showing distribution of pMAMs and dMAMs in advanced metastatic tumors (d10) based on in situ labeling. **b**, Violin plots depicting expression levels of KC (*Vsig4*, *Clec4f* and *Timd4*) and MoM (*Ccr2*) genes in pMAMs and dMAMs. **c**, Representative immunofluorescent images showing distributions of KCs (CD68^+^VSIG4^+^) and MoMs (CD68^+^VSIG4^−^) in tumor core (top) and margin areas (bottom, dashed line) of liver metastasis derived from patients with PDAC (*n* = 3 patients). Metastatic cancer cells were indicated by CK19^+^ staining. Scale bar, 50 µm. **d**, Quantification of KCs and MoMs among intralesional/core macrophages as shown in **c**. Error bars, mean ± s.e.m. *P* value, two-tailed unpaired *t*-test. **e**, UMAP plot identifying six clusters of MoMs derived from cluster 2, 3 and 7 in the original UMAP (Fig. 1c). **f**, Heat map depicting relative expression of upregulated DEGs in each MoM cluster compared to all other MoM clusters in the RNA-seq dataset. Representative genes are labeled for each cluster. **g**, UMAP plots depicting distribution of MoM clusters in early (d5) and advanced metastatic livers (d10). **h**, Diagram showing distribution of MoM clusters in early (d5) and advanced metastatic livers (d10). **i**–**k**, Representative immunofluorescent images of different macrophages (F4/80^+^) expressing antigen presentation marker CD74 or M2 marker YM-1 in early (**i**) and advanced (**j**) liver metastasis derived from experimental intrasplenic model (*n* = 3 mice per group, from one experiment). Scale bars, 50 µm. Quantification of staining showing percentages of intralesional macrophages resembling major MoM clusters: cluster C-like (Cl.C) (CD74^−^YM-1^−^), cluster B-like (Cl.B) (CD74^+^YM-1^−^) and cluster A-like (Cl.A) (CD74^−^YM-1^+^) (**k**). Error bars, mean ± s.e.m. **l**, Relative CD8^+^ T cell activation measured by percentages of interferon (IFN)γ^+^CD8^+^ T cells following stimulation with anti-CD3/CD28-coupled Dynabeads and co-culture with FACS-sorted early or advanced MoMs (F4/80^+^TIM4^−^) from an experimental intrasplenic model compared to a Dynabead-only control (ctrl) (*n* = 3 biological replicates per group from one experiment). Error bars, mean ± s.e.m. *P* values, one-way analysis of variance (ANOVA) with Sidak’s post-test. Source data

**Extended Data Fig. 2:**
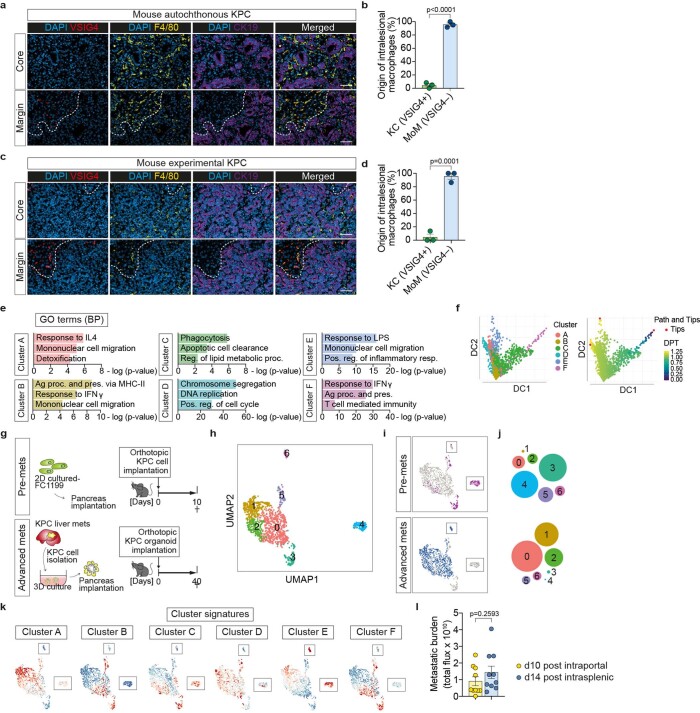
MoMs are proximally located and similar MoM phenotypes are shared in both experimental and spontaneous models of PDAC liver metastasis. **(a)** Representative immunofluorescent images of Kupffer cells (KCs; F4/80^+^VSIG4^+^) and monocyte-derived macrophages (MoMs; F4/80^+^VSIG4^–^) in PDAC liver metastasis from autochthonous KPC model (n = 3 mice, from one experiment). Cancer cells were indicated by CK19^+^ staining. Scale bars, 50 µm. **(b)** Quantification of KCs and MoMs among intralesional/core macrophages (F4/80^+^) in autochthonous KPC model as shown in **(a)**. Error bars, mean ± SEM. *P*-value, two-tailed unpaired t-test. **(c)** Representative immunofluorescent images of KCs (F4/80^+^VSIG4^+^) and MoMs (F4/80^+^VSIG4^–^) in PDAC liver metastasis from experimental intrasplenic KPC model (n = 3 mice, from one experiment). Cancer cells were indicated by CK19^+^ staining. Scale bars, 50 µm. **(d)** Quantification of KCs and MoMs among intralesional/core macrophages (F4/80^+^) in experimental KPC model as shown in **(c)**. Error bars, mean ± SEM. *P*-value, two-tailed unpaired t-test. **(e)** Enriched biological processes (Gene Ontology; GOBP) in MoM clusters identified by g:profiler. **(f)** Diffusion pseudotime graphs of MoM clusters. **(g)** Schematic of generations of pre-metastatic and spontaneous advanced metastatic livers from which MoMs were isolated before subjected to single cell RNA sequencing. **(h)** UMAP plot identifying 7 MoM clusters isolated by flow cytometry from pre-metastatic and advanced metastatic livers induced by orthotopic implantation of KPC-derived cells and organoid, respectively. **(i)** UMAP plots depicting distribution of MoM clusters in pre-metastatic and spontaneous advanced metastatic livers. **(j)** Diagrams showing distribution of MoM clusters in pre-metastatic and spontaneous advanced metastatic livers. **(k)** UMAP plots depicting signature scores of the MoM clusters from experimental liver metastasis models in pre-metastatic and spontaneous advanced metastatic livers. **(l)** Tumor burden of advanced liver metastasis induced by intraportal (d10) or intrasplenic (d14) injections of KPC-derived cells (n = 10 mice/group). Error bars, mean ± SEM. *P*-value, two-tailed unpaired t-test. Source data

We analyzed the spatial distribution of MAM clusters. pMAM and dMAM subsets from advanced liver metastasis displayed minimal overlap, confirming the specificity of our labeling approach (Fig. 2a). We found that metastasis-infiltrating pMAMs originate from monocytes, whereas dMAMs mainly consist of tissue-resident KCs, consistent with a previous report^21^ (Fig. 2b). Tissue section analyses of advanced metastatic lesions derived from patients with PDAC (Fig. 2c,d), an autochthonous KPC mouse model (Extended Data Fig. 2a,b) and an experimental metastasis mouse model (Extended Data Fig. 2c,d), all confirmed that metastatic tumors were highly infiltrated by macrophages. In all cases peripheral macrophages stained positive for the KC marker VSIG4, whereas macrophages within the core were VSIG4-negative. These data suggest that recruited MoMs infiltrate the metastatic lesions, whereas MAMs distal to metastatic lesions originate from tissue-resident KCs.

As pMAMs in advanced metastasis are predominantly derived from monocytes, we performed additional clustering and dimensionality reduction on MoM populations, clusters 2, 3 and 7. We found six further distinct MoM clusters (Fig. 2e). Within the AP-positive-associated subset (original cluster 2), we found three new clusters (B, C and F). Notably, the AP signature was enriched in cluster B and F (AP^high^), but not in cluster C (AP^−/low^) (Fig. 2f). Cluster C was associated with phagocytosis/apoptotic cell-clearance signatures (*Cd300a*, *Cd36*, *Scarb1*, *Anxa2* and *Gpnmb*) and enriched in lysosomal genes (*Lipa*, *Ctsb*, *Ctsd*, *Psap* and *Grn*) (Fig. 2f and Extended Data Fig. 2e), suggestive of efferocytic macrophages. Two clusters (cluster A and E) were derived from the M2-like/immunosuppressive subset (original cluster 3) (Fig. 2f and Extended Data Fig. 2e). Both clusters lacked AP gene expression (AP^−^) and showed higher expressions of the M2 marker genes *Chil3* and *Mrc1*. Cluster E also displayed an inflammatory signature, expressing NF-κB and TNFα signaling-related genes such as *Il6*, *Tnf* and *Il1a* (Fig. 2f and Extended Data Fig. 2e). Cluster D, displayed enrichment of cell-cycle genes and a similar profile to cluster A, suggesting that cluster D is a proliferating subset of cluster A (Fig. 2f and Extended Data Fig. 2e).

In early metastasis, the MoM population was dominated by cluster C, with a minor contribution from the AP^high^-associated subsets clusters B and F. Advanced metastatic lesions were dominated by cluster B and the M2-like subset cluster A, followed by clusters E and D (Fig. 2g,h). Pseudotime analysis revealed a phenotype trajectory that starts with cluster F, followed by cluster C and cluster B, and final differentiation into cluster A/D or E during metastatic progression (Extended Data Fig. 2f). In pre-metastatic livers isolated from mice with orthotopically implanted tumors, we detected AP^high^ and cluster C/E-like MoM populations (Extended Data Fig. 2g–k), showing the presence of cancer-educated MoM populations during pre-metastatic niche formation in tumor-bearing mice. A cluster A-like population was dominant in livers with advanced spontaneous metastasis isolated from mice orthotopically implanted with KPC tumor organoids (Extended Data Fig. 2g–k). These findings were supported by immunostaining of AP protein CD74 and M2-marker *Chil3*-encoded YM-1 in early and advanced metastatic tissues isolated from post-intrasplenic KPC cell implantation. Livers were collected at d5 and d14 (comparable tumor burden as d10 post-intraportal implantation (Extended Data Fig. 2l)). As expected, CD74^−/low^YM-1^−^ MoMs (cluster C) are most abundant in early metastatic lesions, whereas CD74^−/low^YM-1^+^ MoMs (cluster A) represented the most frequent MoM subset in advanced metastatic lesions (Fig. 2i–k).

To characterize the functional phenotype of AP^high^ versus AP^−/low^ MoMs, we isolated CD74^hi^ and CD74^−^^/low^ MoMs (F4/80^+^TIM4^−^) from early and advanced metastatic tumors using FACS and co-cultured them with activated CD8^+^ T cells. In both cases CD74^−/low^ MoMs were more potent in inhibiting CD8^+^ T cell functions compared to CD74^hi^ MoMs (Fig. 2l). We observed lower immunosuppressive activity in CD74^−/low^ MoMs from advanced tumors compared to early CD74^−/low^ MoMs. This may be attributed to the inflammatory subset (cluster E) present in late CD74^−/low^ MoMs (Fig. 2g) that express T cell-stimulating factors such as *Il1a* and *Tnf*^22,23^. Collectively, our data suggest that an immunosuppressive phenotype is acquired in MoMs early during metastasis and is associated with the loss of CD74 expression and the presence of an efferocytosis gene signature.

### Efferocytosis mediates an immunosuppressive phenotype

**Fig. 3:**
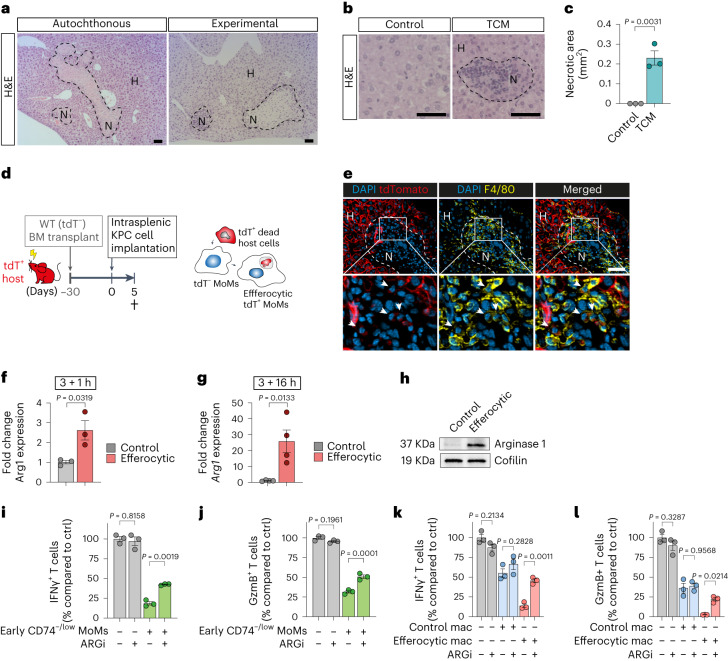
Tissue-resident cell death triggers efferocytosis-mediated immunosuppressive conversion in MoMs. **a**, Representative hematoxylin and eosin (H&E) images of hepatic necroses in autochthonous KPC mice with pre-metastatic PDAC (left) and 48 h post-intrasplenic implantation of KPC cells in WT mice (right). Dotted lines demarcate necrotic areas (N, necrotic; H, healthy) (*n* = 3 mice per group, from one experiment). Scale bars, 50 µm. **b**,**c**, Mice were given KPC TCM or control DMEM once daily for 3 d. Livers were collected 24 h after the last injection. Representative H&E images of livers (**b**) and quantification of hepatic necroses areas (**c**) in the livers (*n* = 3 mice per group, from one experiment). Dotted line demarcates the necrotic area. Scale bars, 50 µm. Error bars, mean ± s.e.m. *P* value, two-tailed unpaired *t*-test. **d**, Schematic of chimeric mice generation via transplantation of non-labeled (tdT^−^) donor BM cells into tdT^+^ hosts (*n* = 3 mice). **e**, Representative immunofluorescent images of efferocytic MoMs (arrowheads, tdT^+^ debris-containing F4/80^+^tdT^−^ cells) in healing necrotic areas (*n* = 3 mice, from one experiment). Scale bar, 50 µm. **f**,**g**, qPCR analysis of *Arg1* in BMMs co-cultured for 3 h with apoptotic thymocytes at 1 h (**f**) or 16 h (**g**) after washout. BMM, bone marrow-derived macrophages. Error bars, mean ± s.e.m. (*n* = 3 biological replicates per group). *P* value, two-tailed unpaired *t*-test. **h**, Representative western blot image of arginase 1 and loading control cofilin levels in BMMs (3 + 16 h, experiment was performed three times with similar results). **i**,**j**, Relative activation levels of CD8^+^ T cells, measured as percentages of IFNγ^+^ (**i**) or granzyme B (GzmB)^+^ (**j**), stimulated with anti-CD3/CD28-coupled Dynabeads and co-cultured with CD74^−/low^ MoMs (F4/80^+^TIM4^−^) from d5 livers compared to Dynabead-only control (*n* = 3 biological replicates per group from one experiment). Error bars, mean ± s.e.m. *P* values, one-way ANOVA with Sidak’s post-test. **k**,**l**, Relative activation levels of CD8^+^ T cells, measured as percentages of IFNγ^+^ (**k**) or granzyme B (GzmB)^+^ cells (**l**), stimulated with anti-CD3/CD28-coupled Dynabeads and co-cultured with BMMs compared to Dynabead-only control (*n* = 3 biological replicates per group). Error bars, mean ± s.e.m. *P* values, one-way ANOVA with Sidak’s post-test. Source data

**Extended Data Fig. 3:**
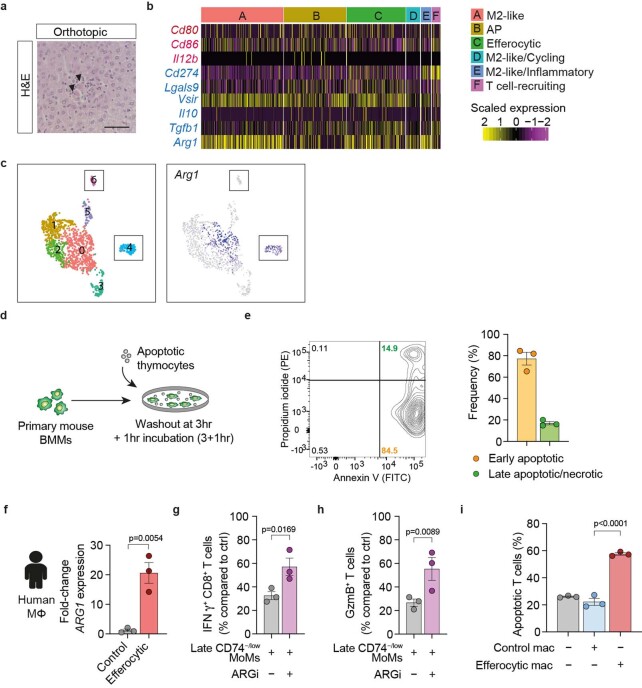
Efferocytosis induces immunosuppressive activity in macrophages that is mediated by Arginase 1. **(a)** Representative H&E image of dead hepatocytes in pre-metastatic liver at d10 post pancreatic implantation of KPC-derived cells (n = 3 mice, from one experiment). Scale bar, 50 µm. **(b)** Heatmap depicting relative expression of T cell-stimulatory (red) or repressive genes (blue) in MoM clusters. **(c)** UMAP plots depicting *Arg1* expression levels in MoMs isolated from pre-metastatic and spontaneous advanced metastatic livers. **(d)** Schematic of efferocytosis assay in primary bone marrow-derived macrophages (BMMs). **(e)** FACS plot (left) and quantification (right) of apoptotic thymocytes post staurosporine treatment (n = 3 biological replicates/group). Error bars, mean ± SEM. **(f)** qPCR analysis of *ARG1* in human primary MoMs (n = 3 biological replicates/group). Error bars, mean ± SEM. *P*-value, two-tailed unpaired t-test. **(g,h)** Relative activation levels of CD8^+^ T cell, measured as percentages of IFNγ^+^ **(g)** or GzmB^+^ **(h)** cells, stimulated with anti-CD3/CD28-coupled Dynabeads and co-cultured with MoMs from advanced metastatic livers compared to Dynabeads-only control (n = 3 biological replicates/group from one experiment). Error bars, mean ± SEM. *P*-values, one-way ANOVA with Sidak’s post-test. **(i)** Percentages of apoptotic (Apotracker^+^) CD8^+^ T-cells, following stimulation with anti-CD3/CD28-coupled Dynabeads and co-culture with BMMs (n = 3 biological replicates/group). Error bars, mean ± SEM. *P*-values, one-way ANOVA with Sidak’s post-test. Source data

We reasoned that the observed pathway signatures in MoMs might be induced by metastasis-related liver injury. To test this, we examined the extent of metastasis-related injury in livers derived from (1) an autochthonous KPC model, (2) an experimental intrasplenic metastasis model and (3) after daily intravenous administration of pancreatic tumor-conditioned medium (TCM) into healthy mice. Hepatic necroses were detected in the liver tissues of the autochthonous KPC model, post-orthotopic implantation of KPC cells and at the early stages of the experimental intrasplenic metastasis model (Fig. 3a and Extended Data Fig. 3a). Daily administration of pancreatic TCM was sufficient to induce hepatic necroses (Fig. 3b,c). Thus, PDAC liver metastasis is accompanied by resident cell death during initial metastatic spreading, and even in response to, tumor-derived factors. To confirm the presence of efferocytic MoMs, we transplanted wild-type (WT) bone-marrow (BM) cells into tdTomato-expressing (tdT^+^) mice, resulting in chimeric mice that have ubiquitous expression of tdT, including in hepatocytes and KCs, whereas MoMs remained unlabeled (Fig. 3d). Following induction of liver metastasis, we observed accumulation of tdT^−^ MoMs in necrotic areas, some of which contained tdT^+^ debris in their cytoplasm (Fig. 3e). These data show that an efferocytotic MoM subpopulation emerges early in liver metastasis to resolve tissue injuries.

To identify the mediator of immunosuppressive activity in the early MoM subset, we examined expression of immunosuppressive genes in the MoM population in our scRNA-seq data. Similar to M2-like clusters A/D/E, we found T cell inhibitory gene *Arg1* expression in cluster C MoMs, which may be responsible for the immunosuppressive activity of early CD74^−/low^ MoMs (Extended Data Fig. 3b). Furthermore, arginase 1 has been linked to efferocytosis^24,25^. Consistent with the presence of liver injury before metastatic engraftment, high *Arg1* expression was found in MoM populations isolated from pre-metastatic livers of mice with orthotopically implanted tumor (Extended Data Fig. 3c). In vitro cell culture assays using primary mouse and human macrophages confirmed that efferocytosis is sufficient to induce the upregulation of *Arg1*/arginase 1 (Fig. 3f–h and Extended Data Fig. 3d–f).

To test the biological relevance of arginase 1, we stimulated CD8^+^ T cells and co-cultured them with CD74^−/low^ early MoMs (Fig. 3i, j) or efferocytic macrophages (Fig. 3k,l) in the presence of an arginase 1 inhibitor, CB1158. Similar to early MoMs, efferocytic macrophages showed markedly increased immunosuppressive activity and treatment with arginase 1 inhibitor CB1158 (ARGi) abrogated this effect (Fig. 3i–l). This suggests that arginase 1 is responsible for the T cell-suppressing activity of efferocytic macrophages. Blockade of arginase 1 activity also reduced the T cell inhibitory effect of CD74^−/low^ late MoMs, suggesting that arginase 1 mediates immunosuppressive effects in both early and late CD74^−/low^ MoMs (Extended Data Fig. 3g,h). We also observed increased apoptosis of stimulated CD8^+^ T cells when co-cultured with efferocytic macrophages in vitro (Extended Data Fig. 3i). In summary, PDAC liver metastasis is accompanied by liver damage and efferocytosis, promoting the conversion of macrophages toward an immunosuppressive phenotype.

### Inhibiting efferocytosis halts MoM conversion and metastasis

**Fig. 4:**
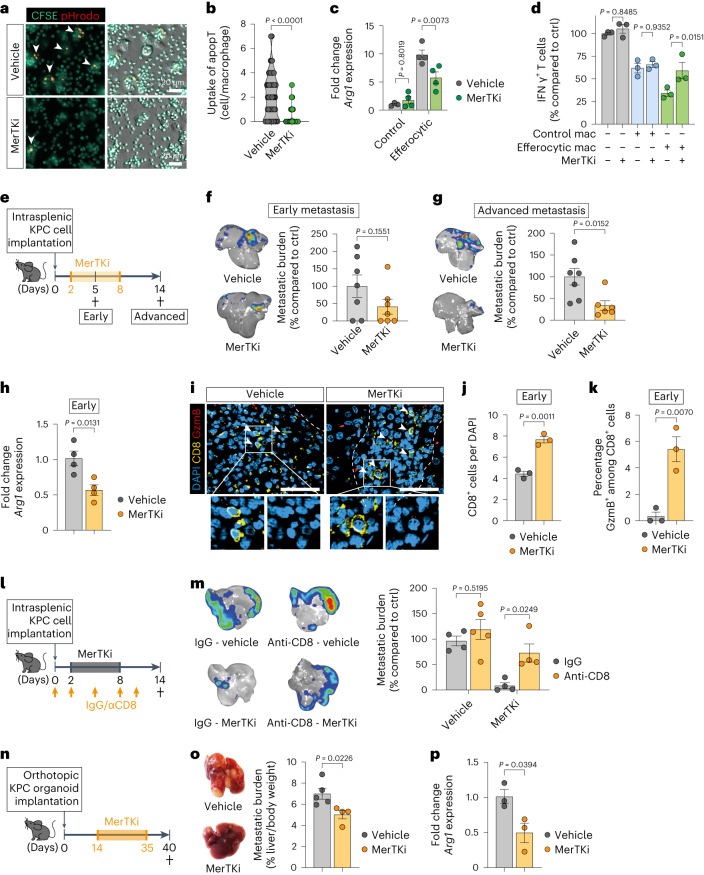
Inhibition of efferocytosis prevents MoM conversion and PDAC metastasis. **a**,**b**, Representative fluorescence image (**a**) and quantification of engulfed CSFE/pHrodo-labeled apoptotic thymocytes in BMMs (**b**) (vehicle *n* = 65 cells; MerTKi *n* = 112 cells, experiment was performed twice with similar results). CFSE, carboxyfluorescein succinimidyl ester. Error bars, mean ± s.e.m. *P* value, two-tailed unpaired *t*-test. **c**, qPCR analysis of *Arg1* in BMMs (20 h). Error bars, mean ± s.e.m. (control/vehicle *n* = 3, control/MerTKi *n* = 4, efferocytic/vehicle *n* = 4, efferocytic/MerTKi *n* = 4 biological replicates). *P* values, two-way ANOVA with Sidak’s post-test. **d**, Relative activation level of CD8^+^ T cell, measured as percentages of IFNγ^+^ cells, stimulated with anti-CD3/CD28-coupled Dynabeads and co-cultured BMMs compared to Dynabead-only control (*n* = 3 biological replicates per group). Error bars, mean ± s.e.m. *P* values, two-way ANOVA with Sidak’s post-test. **e**, Schematic illustrating the MerTKi experiment timeline. **f**,**g**, Representative bioluminescence imaging (BLI) images (left) and relative tumor burden (right) of d5 (*n* = 7 mice per group, from one experiment) (**f**) or d14 livers (**g**) (control *n* = 7 mice, MerTKi *n* = 6 mice, from two experiments). Error bars, mean ± s.e.m. *P* values, two-tailed unpaired *t*-test. **h**, qPCR analysis of *Arg1* in MoMs (F4/80^+^TIM4^−^) from d5 livers (*n* = 4 mice per group). Error bars, mean ± s.e.m. *P* value, two-tailed unpaired *t*-test. **i**–**k**, Representative immunofluorescence images (**i**) and quantification of total (**j**) and cytotoxic GzmB^+^ (**k**) CD8^+^ T cells in d5 livers. Arrowheads indicate CD8^+^ T cells (*n* = 3 mice per group, from one experiment). Scale bars, 50 µm. Error bars, mean ± s.e.m. *P* values, two-tailed unpaired *t*-test. **l**, Schematic illustrating the CD8^+^ T cell depletion experiment timeline. **m**, Representative ex vivo BLI images (left) and relative tumor burden (right) of d14 livers (vehicle/IgG *n* = 4 mice, vehicle/anti-CD8 *n* = 5 mice, MerTKi/IgG *n* = 4 mice, MerTKi/anti-CD8 *n* = 4 mice, from one experiment). Error bars, mean ± s.e.m. *P* values, two-way ANOVA with Sidak’s post-test. **n**, Schematic diagram illustrating the MerTKi experiment in spontaneous liver metastasis model. **o**, Representative liver photographs (left) and d40 tumor burden from indicated treatment groups (control *n* = 5 mice, MerTKi *n* = 4 mice, from one experiment). Error bars, mean ± s.e.m. *P* value, two-tailed unpaired *t*-test. **p**, qPCR analysis of *Arg1* in MoMs (F4/80^+^TIM4^−^) from d40 livers (*n* = 3 mice per group). Error bars, mean ± s.e.m. *P* value, two-tailed unpaired *t*-test. Source data

**Extended Data Fig. 4:**
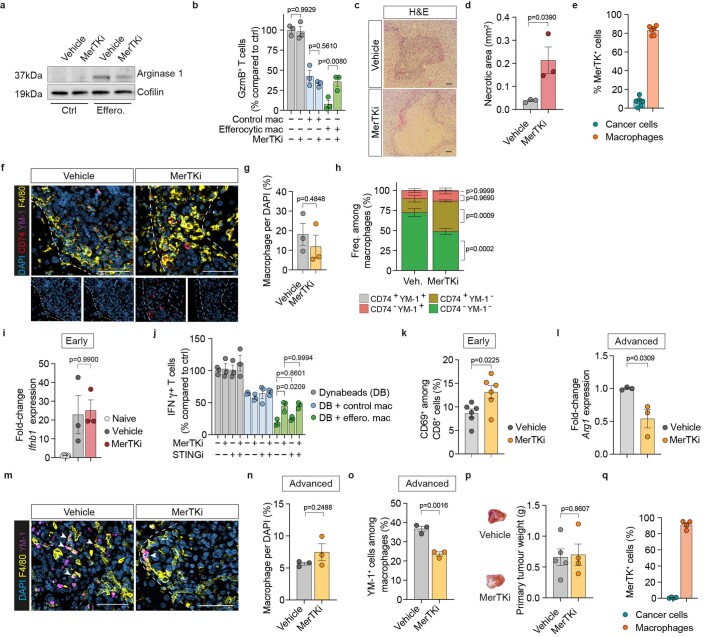
Efferocytosis induces immunosuppressive macrophage conversion. **(a**) Representative Western blot image of Arginase 1 and loading control Cofilin levels in BMMs (experiment was performed three times with similar results). **(b)** Relative activation levels of CD8^+^ T cell, measured as percentages of IFNγ^+^ cells, stimulated with anti-CD3/CD28-coupled Dynabeads and co-cultured with d5 liver MoMs (F4/80^+^TIM4^–^) compared to Dynabeads-only control (n = 3 biological replicates/group from one experiment). Error bars, mean ± SEM. *P*-values, one-way ANOVA with Sidak’s post-test. **(c,d)** Representative H&E images **(c)** and quantification **(d)** of necroses in d5 livers (n = 3 mice/group, from one experiment). Scale bars, 50 µm. Error bars, mean ± SEM. *P*-value, two-tailed unpaired t-test. **(e)** Flow cytometry analysis of MerTK^+^ cells among cancer cells (CD45^–^ZsGreen+) or macrophages (CD45^+^F4/80^+^) in d14 livers (n = 6 mice/group). Error bars, mean ± SEM. **(f-h)** Representative immunofluorescence images **(f)** and quantification of total **(g)** and CD74^−^ and/or YM-1-expressing **(h)** macrophages (F4/80^+^) in d5 livers (n = 3 mice/group, from one experiment). Scale bars, 50 µm. Error bars, mean ± SEM. *P*-value, two-tailed unpaired t-test. **(i**) qPCR analysis of *Ifnb* in MoMs (F4/80^+^TIM4^–^) from d5 livers (n = 3 mice/group). Error bars, mean ± SEM. *P*-value, two-tailed unpaired t-test. **(j)** Relative activation levels of CD8^+^ T cells, measured as percentages of IFNγ^+^ cells, stimulated with anti-CD3/CD28-coupled Dynabeads and co-cultured with d5 liver MoMs (F4/80^+^TIM4^–^) compared to Dynabeads-only control (n = 3 biological replicates/group from one experiment). Error bars, mean ± SEM. *P*-values, one-way ANOVA with Sidak’s post-test. **(k)** Flow cytometry analysis of activated (CD69^+^) CD8^+^ T cells in d5 livers (n = 6 mice/group). Error bars, mean ± SEM. *P*-value, two-tailed unpaired t-test. **(l)** qPCR analysis of *Arg1* in MoMs (F4/80^+^TIM4^–^) isolated from d14 livers (n = 3 mice/group). Error bars, mean ± SEM. *P*-value, two-tailed unpaired t-test. **(m-o)** Representative immunofluorescence images **(m)** and quantification of total **(n)** and YM-1^+^ **(o)** macrophages (F4/80^+^) in d14 livers. Arrowheads indicate YM-1^+^ macrophages (n = 3 mice/group, from one experiment). Scale bars, 50 µm. Error bars, mean ± SEM. *P*-value, two-tailed unpaired t-test. **(p)** Organoid-derived primary tumor burden at d40. Error bars, mean ± SEM. *P*-value, two-tailed unpaired t-test. **(q)** Flow cytometry analysis of MerTK^+^ cells among cancer cells (CD45^–^Epcam+) or macrophages (CD45^+^F4/80^+^) in d40 livers (n = 6 mice/group). Error bars, mean ± SEM. Source data

To assess the biological function of efferocytosis-induced macrophage conversion in PDAC liver metastases, we blocked efferocytosis in vitro using an inhibitor of tyrosine protein kinase Mer (MerTK), UNC2250 (MerTKi). As expected, MerTKi reduced efferocytosis in macrophages co-cultured with apoptotic thymocytes (Fig. 4a,b) and ablated the induction of *Arg1* expression in macrophages (Fig. 4c and Extended Data Fig. 4a). Furthermore, MerTKi reduced the T cell suppressing activity of efferocytic macrophages (Fig. 4d and Extended Data Fig. 4b).

To test these findings in vivo, we induced liver metastasis by intrasplenic implantation of KPC cells, followed by treatment of the animals with MerTKi for seven consecutive days (Fig. 4e). Hepatic necrotic areas were markedly increased in early metastatic lesions (d5) in livers derived from mice treated with MerTKi, suggesting a delay in clearing apoptotic cells due to impaired efferocytosis (Extended Data Fig. 4c,d). MerTKi administration did not affect early metastatic tumor burden; however, a significant reduction was found at the advanced stage (Fig. 4f,g).

While some cancer cells express MerTK^26–28^, we found that only a low percentage of the KPC-derived cell line FC1199 express this receptor (Extended Data Fig. 4e), suggesting that MerTKi does not act on cancer cell function. In contrast, the majority of macrophages expressed MerTK (Extended Data Fig. 4e). Analysis of isolated MoMs confirmed a significant reduction in *Arg1* expression in early metastatic lesions when efferocytosis is impaired (Fig. 4h). While MoM abundance was not affected, MerTKi treatment caused a decrease in CD74^−^YM-1^−^ and an increase in CD74^+^YM-1^−^ MoMs (Extended Data Fig. 4f–h).

MerTK inhibition has been shown to activate the STING/type I interferon pathway^29^. We observed increased *Ifnb1* among early MoMs; however, this was not affected by MerTK inhibition (Extended Data Fig. 4i). Moreover, while MerTKi ablated the immunosuppressive activities of efferocytic macrophages, additional pharmacological blockade of STING did not show any effect, suggesting a dispensable role of the STING/type I interferon pathways in this setting (Extended Data Fig. 4j).

In addition to a greater abundance of less-immunosuppressive macrophages in early metastatic lesions of MerTKi-treated mice, we found a significant increase in infiltrating CD8^+^ T cells (Fig. 4i,j). Among these we saw a significantly higher proportion of cytotoxic GzmB^+^CD8^+^ T cells (Fig. 4i,k) and activated CD69^+^CD8^+^ T cells (Extended Data Fig. 4k). Depletion of CD8^+^ T cells ablated the anti-metastatic effect of MerTK treatment (Fig. 4l,m) suggesting that MerTKi acts through increasing cytotoxic T cell functions.

Of note, even in advanced metastatic tumors (d14), MerTKi treatment reduced *Arg1* expression in MoMs (Extended Data Fig. 4l) without affecting overall abundance of MoMs (Extended Data Fig. 4m,n). Furthermore, MerTKi-treated lesions contained fewer YM-1^+^ macrophages (Extended Data Fig. 4m,o), confirming that inhibition of efferocytosis in early metastasis impairs the phenotypic conversion of MoMs during metastatic progression in vivo.

Similar reduction in metastatic tumor burden was found with MerTK inhibition in the spontaneous liver metastasis model (Fig. 4n,o), whereas primary tumors remained unaffected (Extended Data Fig. 4p). There was no MerTK expression in the KPC-derived organoid used in this model (Extended Data Fig. 4q). Expectedly, *Arg1* expression in MoMs was reduced with MerTKi treatment (Fig. 4p).

**Extended Data Fig. 5:**
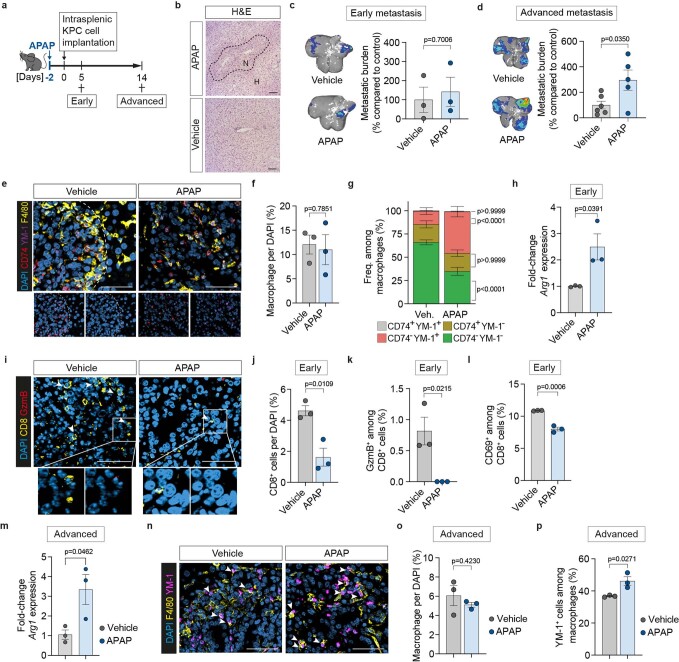
Liver injury facilitates liver metastasis and induces immunosuppressive macrophage conversion. **(a)** Schematic diagram illustrating the APAP experiment timeline. **(b)** Representative H&E images of hepatic necroses at 24 hours post vehicle or APAP injection (n = 3 mice/group, from one experiment). Scale bars, 50 µm. **(c,d)** Representative *ex vivo* bioluminescence imaging (BLI) images (left) and relative tumor burden (right) of d5 **(c)** and d14 **(d)** livers (d5 n = 3 mice/group, from one experiment; d14 control n = 6 mice; APAP n = 5 mice, from two experiments). Error bars, mean ± SEM. *P*-value, two-tailed unpaired t-test. **(e-g)** Representative immunofluorescence images **(e)** and quantification of total (**f**) and CD74^−^ and/or YM-1-expressing **(g)** macrophages (F4/80^+^) in d5 livers (n = 3 mice/group). Scale bars, 50 µm. Error bars, mean ± SEM. *P*-value, two-tailed unpaired t-test. **(h)** qPCR analysis of *Arg1* in MoMs (F4/80^+^TIM4^–^) from d5 livers (n = 3 mice/group). Error bars, mean ± SEM. *P*-value, two-tailed unpaired t-test. **(i-k)** Representative immunofluorescence images **(i)** and quantification of total **(j)** and cytotoxic granzyme B^+^ **(k)** CD8^+^ T-cells in d5 livers (n = 3 mice/group). Arrowheads indicate CD8^+^ T cells. Scale bars, 50 µm. Error bars, mean ± SEM. *P*-value, two-tailed unpaired t-test. **(l)** Flow cytometry analysis of activated (CD69^+^) CD8^+^ T cells in d5 livers (n = 3 mice/group). Error bars, mean ± SEM. *P*-value, two-tailed unpaired t-test. **(m)** qPCR analysis of *Arg1* in MoMs (F4/80^+^TIM4^–^) from d14 livers (n = 3 mice/group). Error bars, mean ± SEM. *P*-value, two-tailed unpaired t-test. **(n-p)** Representative immunofluorescence images **(n)** and quantification of total **(o)** and YM-1^+^ **(p)** macrophages (F4/80^+^) (n = 3 mice/group). Arrowheads indicate YM-1^+^ macrophages. Scale bars, 50 µm. Error bars, mean ± SEM. *P*-value, two-tailed unpaired t-test. Source data

Metastasis-associated liver injury induces a pro-tumorigenic MAM phenotype, we therefore questioned whether non-cancerous liver injury increases the presence of pro-tumorigenic MAMs, making livers more permissive for metastasis. We induced acute liver injury using a single dose of paracetamol (*N*-acetyl-para-aminophenol; APAP). Two days later, animals underwent intrasplenic implantation of pancreatic cancer cells (Extended Data Fig. 5a). As expected^30,31^, APAP induced hepatic necroses 24 h after administration (Extended Data Fig. 5b). APAP pre-treatment showed marginal effect on early metastatic outgrowth (Extended Data Fig. 5c), but resulted in significantly increased tumor burden at advanced stage compared to vehicle pretreatment (Extended Data Fig. 5d). APAP pretreatment did not affect CD74^+^YM-1^−^ MoMs in early metastatic lesions (Extended Data Fig. 5e–g) but reduced the proportion of CD74^−^YM-1^−^ MoMs and increased CD74^−^YM-1^+^ MoMs (Extended Data Fig. 5e–g), indicating accelerated MoM conversion into the late-stage M2-like phenotype. Expression of *Arg1* in MoMs was reduced with APAP pretreatment compared to vehicle-treated animals (Extended Data Fig. 5h), whereas CD8^+^ T cell infiltration (Extended Data Fig. 5i,j) and their cytotoxicity/activation state (Extended Data Fig. 5i,k,l) were significantly reduced. Advanced metastatic lesions in APAP pretreated mice showed sustained significant elevated *Arg1* expression (Extended Data Fig. 5m), whereas MoM numbers were unchanged (Extended Data Fig. 5n,o). An increased proportion of YM-1^+^ macrophages was found compared to that in vehicle control-derived livers (Extended Data Fig. 5n,p).

These findings demonstrate that an efferocytosis-induced macrophage switch promotes PDAC liver metastasis and that this process can be blocked by inhibiting MerTK.

### Progranulin deletion reduces PDAC liver metastasis

**Fig. 5:**
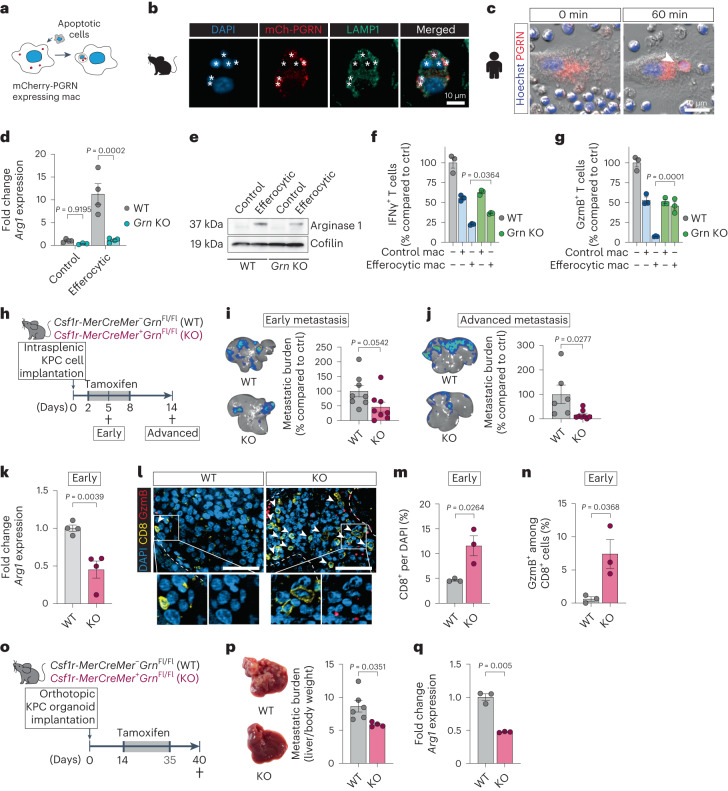
Depletion of progranulin prevents macrophage conversion and reduces PDAC liver metastasis. **a**, Schematic illustrating experiment to track progranulin localization in efferocytic mCherry-PGRN-expressing macrophages. PGRN, progranulin. **b**, Representative immunofluorescence image of mCherry-PGRN in efferosomes (asterisks) of murine BMMs (*n* = 33 cells, experiment was performed twice with similar results). Scale bar, 50 µm. **c**, Representative fluorescence image of mCherry-PGRN in efferosome (arrowhead) of human MoMs over time as assessed by live-cell imaging (experiment was performed twice with similar results). **d**, qPCR analysis of *Arg1* in BMMs (20 h, *n* = 4 biological replicates per group). Error bars, mean ± s.e.m. *P* values, two-way ANOVA with Sidak’s post-test. **e**, Representative western blot image of arginase 1 and loading control cofilin levels in BMMs (3 + 16 h, experiment was performed three times with similar results). **f**,**g**, Relative activation of CD8^+^ T cell, measured by percentages of IFNγ^+^ (**f**) or granzyme B (GzmB)^+^ (**g**) T cells, stimulated with anti-CD3/CD28-coupled Dynabeads and co-cultured with BMMs compared to Dynabead-only control (*n* = 3 biological replicates per group from one experiment). Error bars, mean ± s.e.m. *P* values, one-way ANOVA with Sidak’s post-test. **h**, Schematic illustrating the *Grn* KO experiment timeline. **i**,**j**, Representative ex vivo BLI images (left) and relative tumor burden (right) of d5 (*n* = 8 mice per group, from two experiments) (**i**) and d14 (WT, *n* = 6 mice; KO, *n* = 8 mice, from two experiments) (**j**) livers. Error bars, mean ± s.e.m. *P* values, two-tailed unpaired *t*-test. **k**, qPCR analysis of *Arg1* in MoMs (F4/80^+^TIM4^−^) from d5 livers (*n* = 4 mice per group). Error bars, mean ± s.e.m. *P* values, two-tailed unpaired *t*-test. **l**–**n**, Representative immunofluorescence images (**l**) and quantification of total (**m**) and cytotoxic GzmB^+^ (**n**) CD8^+^ T cells in d5 livers (*n* = 3 mice per group, from one experiment). Arrowheads indicate CD8^+^ T cells. Scale bars, 50 µm. Error bars, mean ± s.e.m. *P* values, two-tailed unpaired *t*-test. **o**, Schematic illustrating the *Grn* KO experiment in a spontaneous liver metastasis model. **p**, Representative liver photographs (left) and tumor burden (WT, *n* = 6 mice; KO, *n* = 4 mice, from one experiment). Error bars, mean ± s.e.m. *P* values, two-tailed unpaired *t*-test. **q**, qPCR analysis of *Arg1* in MoMs (F4/80^+^TIM4^−^) from d5 livers (*n* = 3 mice per group). Error bars, mean ± s.e.m. *P* values, two-tailed unpaired *t*-test. Source data

**Extended Data Fig. 6:**
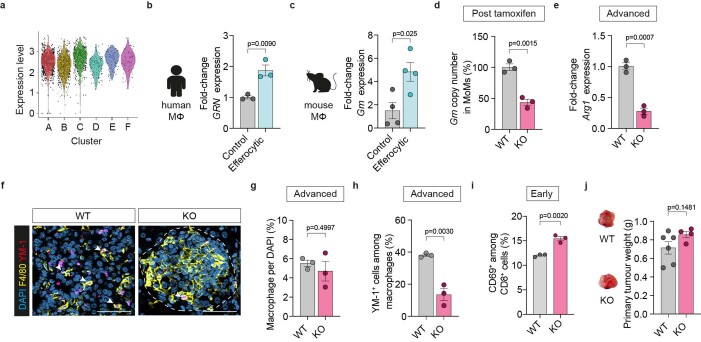
Progranulin mediates efferocytosis-induced macrophage conversion. **(a)** Violin plot depicting expression of *Grn* in MoM clusters from the scRNAseq dataset. **(b)** qPCR analysis of *GRN* in THP-1-derived macrophages (20hrs). Error bars, mean ± SEM (n = 3 independent experiments). *P*-value, two-tailed unpaired t-test. **(c)** qPCR analysis of *Grn* in murine primary BMMs (20hrs). Error bars, mean ± SEM (n = 4 biological replicates/group). *P*-value, two-tailed unpaired t-test. **(d)** qPCR analysis of *Grn* gene copy number in MoMs (F4/80^+^TIM4^–^) (n = 3 mice/group). Error bars, mean ± SEM. *P*-value, two-tailed unpaired t-test. **(e)** qPCR analysis of *Arg1* in MoMs (F4/80^+^TIM4^–^) from d14 livers (n = 3 mice/group). Error bars, mean ± SEM. *P*-value, two-tailed unpaired t-test. **(f-h)** Representative immunofluorescence images **(f)** and quantification of total **(g)** and YM-1^+^ **(h)** macrophages (F4/80^+^) in d14 livers. Arrowheads indicate YM-1^+^ macrophages (n = 3 mice/group, from one experiment). Scale bars, 50 µm. Error bars, mean ± SEM. *P*-value, two-tailed unpaired t-test. **(i)** Flow cytometry analysis of (CD69^+^) CD8^+^ T-cells in d5 livers (n = 3 mice/group). Error bars, mean ± SEM. *P*-value, two-tailed unpaired t-test. **(j)** Organoid-derived primary tumor burden at d40. Error bars, mean ± SEM. *P*-value, two-tailed unpaired t-test. Source data

Blocking the clearance of necrotic cells leads to increased tissue damage. Interfering with post-engulfment stages of efferocytosis may therefore be a better strategy to suppress macrophage polarization during PDAC liver metastasis. To identify a suitable macrophage-specific target, we analyzed upregulated lysosomal genes in early cluster 2 MoM from our scRNA-seq analysis. Among the upregulated genes was progranulin (*Grn*) (Extended Data Fig. 6a), a precursor protein directed to the lysosomal compartment, where proteolytically cleaved fragments called granulins are thought to be critical for lysosome function in macrophages^32^. We have shown that macrophages are a major source of progranulin in PDAC liver metastasis^9^ and that depletion of progranulin is associated with defective phagocytic activities in response to bacterial infections^33^. We hypothesized that progranulin might play a critical role in macrophage-mediated efferocytosis. In agreement with our hypothesis, we found that efferocytosis induces the expression of *Grn* in primary human and mouse macrophages (Extended Data Fig. 6b,c) and that progranulin localizes to the lysosomal compartment in these cells during efferocytosis (Fig. 5a–c). In addition, efferocytosis-induced upregulation of *Arg1* expression (Fig. 5d,e) and suppression of CD8^+^ T cell function were significantly reduced in progranulin-deficient (*Grn* knockout (KO)) macrophages (Fig. 5f,g).

We tested whether macrophage-specific depletion of progranulin affects MAM polarization during PDAC liver metastasis in vivo. We intrasplenically implanted pancreatic cancer cells into conditional *Csf1r-MerCreMer*^+^;*Grn*^fl/fl^ mice (KO) with *Csf1r-MerCreMer*^–^;*Grn*^fl/fl^ (WT) as a control cohort. In the KO strain, tamoxifen administration induces depletion of progranulin in macrophages. (Fig. 5h and Extended Data Fig. 6d). Macrophage-specific depletion of progranulin significantly reduced metastatic tumor burden in the advanced stage (Fig. 5i,j). Depletion of progranulin reduced expression of *Arg1* in isolated MoMs (Fig. 5k). This reduction was sustained in advanced metastasis (Extended Data Fig. 6e) along with reduction in YM-1^+^ macrophages, whereas macrophage abundance remained unaffected (Extended Data Fig. 6f–h). Lack of progranulin in macrophages led to a significant increase in total CD8^+^ T cell numbers (Fig. 5l,m), in cytotoxic GzmB^+^CD8^+^ T cells (Fig. 5l,n) and in activated CD69^+^CD8^+^ T cells (Extended Data Fig. 6i) in early metastatic livers.

Depletion of progranulin in macrophages also resulted in reduced tumor burden in the spontaneous liver metastasis model without significant change in the primary tumors (Fig. 5o,p and Extended Data Fig. 6j). Consistently, *Arg1* expression in MoMs was also significantly diminished in this model (Fig. 5q).

These data demonstrate that progranulin expression in macrophages is necessary for efferocytosis-induced conversion of MoMs into an immunosuppressive phenotype that then supports metastatic growth of pancreatic cancer cells in the liver.

### Progranulin deficiency impairs lysosomal acidification

**Fig. 6:**
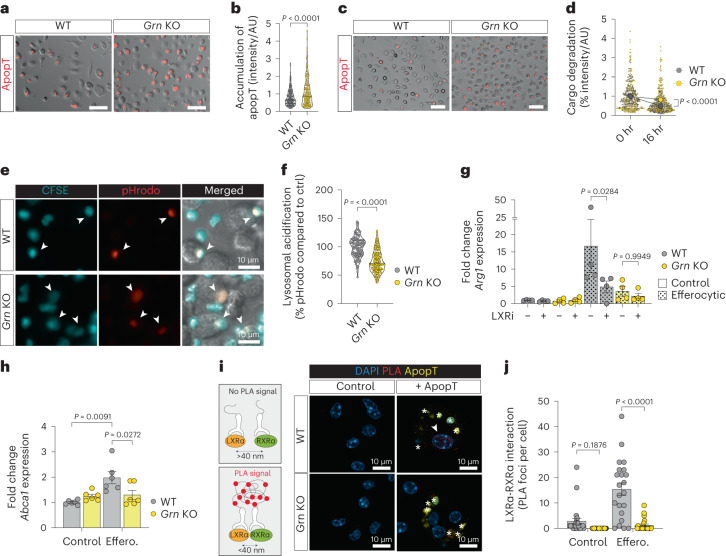
Progranulin deficiency impairs lysosomal acidification and cargo degradation during efferocytosis. **a**,**b**, Representative fluorescence images (**a**) and quantification (**b**) of uptake of CellTrace-labeled apoptotic thymocytes (ApopT) in BMMs (WT, *n* = 223 cells; *Grn* KO, *n* = 240 cells; experiment was performed twice with similar results). Error bars, mean ± s.e.m. *P* value, two-tailed unpaired *t*-test. Scale bars, 50 µm. AU, arbitrary unit. **c**, Representative images of efferocytic BMMs (WT, 0/16 h *n* = 167/200 cells; *Grn* KO, 0/16 h *n* = 220/256 cells; experiment was performed twice with similar results) 16 h after washout of CellTrace-labeled apoptotic thymocytes (ApopT). Scale bars, 50 µm. **d**, Quantification of apoptotic thymocyte cargo degradation in efferocytic BMMs depicted in **c**. Error bars, mean ± s.e.m. *P* value, two-tailed unpaired *t*-test. **e**,**f**, Representative fluorescence images (**e**) and quantification (**f**) of peak pHrodo intensity in BMMs incubated with CFSE/pHrodo-labeled apoptotic thymocytes (WT, *n* = 115 cells; *Grn* KO, *n* = 164 cells; experiment was performed three times with similar results). Error bars, mean ± s.e.m. *P* value, two-tailed unpaired *t*-test. **g**, qPCR analysis of *Arg1* in BMMs (20 h). Error bars, mean ± s.e.m. (*n* = 4 biological replicates per group). *P* values, two-way ANOVA with Sidak’s post-test. **h**, qPCR analysis of LXRα target gene *Abca1* in BMMs (20 h). Error bars, mean ± s.e.m. (*n* = 6 biological replicates per group). *P* values, two-way ANOVA with Tukey’s post-test. **i**, Schematic of proximity ligation assay (PLA) for LXRα and RXRα (left) and representative fluorescence images (right) of PLA probe-bound fluorophore (PLA) in BMMs after incubation with CFSE-labeled apoptotic thymocytes (ApopT, green). Asterisks indicate phagocytosed apoptotic cells. **j**, Quantification of PLA foci (arrowhead) in WT or *Grn* KO BMMs as depicted in **i** (control WT, *n* = 24 cells; control *Grn* KO, *n* = 32 cells; efferocytic WT, *n* = 22 cells; efferocytic *Grn* KO, *n* = 22 cells; experiment was repeated three times with similar results). Error bars, mean ± s.e.m. *P* values, two-way ANOVA with Sidak’s post-test. Source data

**Extended Data Fig. 7:**
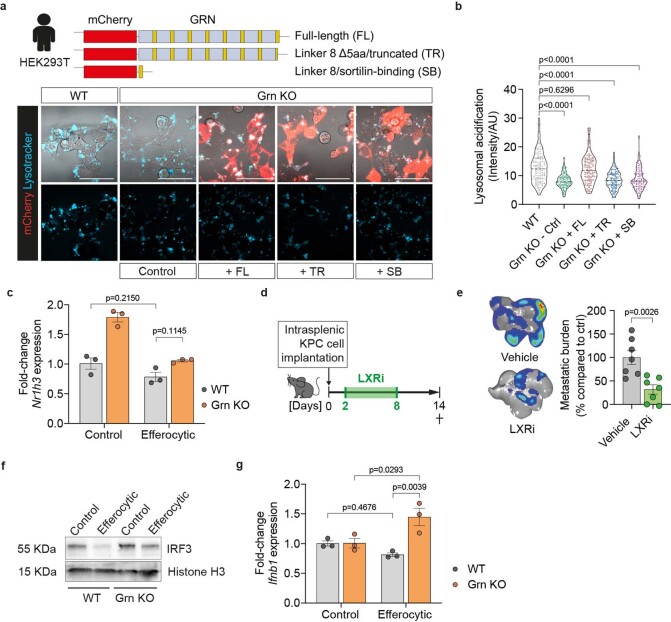
Progranulin deficiency impairs lysosomal acidification. **(a)** Schematic of mCherry-mPGRN constructs (upper) and representative fluorescence images (lower) of transduced, LysoSensor-labeled HEK293T cells (WT n = 58 cells; *GRN* KO n = 60 cells, experiment was performed twice with similar results). Scale bars, 50 µm. **(b)** Quantification of LysoSensor intensity as depicted in **(a)**. Error bars, mean ± SEM. *P*-values, one-way ANOVA with Dunnet’s post-hoc test. **(c)** qPCR analysis of LXRα-encoding gene *Nr1h3* in BMMs (20hrs). Error bars, mean ± SEM (n = 3 biological replicates/group). *P*-values, two-way ANOVA with Sidak’s post-test. **(d)** Schematic diagram illustrating the LXRi experiment timeline. **(e)** Representative *ex vivo* bioluminescence imaging (BLI) images (left) and relative tumor burden (right) of d14 livers (n = 7 mice/group, from one experiment). Error bars, mean ± SEM. *P*-value, two-tailed unpaired t-test. **(f)** Representative Western blot image of IRF3 and loading control Histone H3 levels in nuclear fractions of BMMs following 4^−^hour incubation with apoptotic thymocytes (experiment was performed three times with similar results). **(g)** qPCR analysis of *Ifnb1* in BMMs following 4-hour incubation with apoptotic thymocytes (n = 3 biological replicates/group). Error bars, mean ± SEM. *P*-value, two-way ANOVA with Sidak’s post-test. Source data

To better understand the biological function of progranulin, we tested whether lack of progranulin affects efferocytosis of apoptotic cells and/or their proteolytic degradation in the lysosomes. Notably, progranulin-deficient macrophages (*Grn* KO) showed an increased accumulation of engulfed apoptotic cells (Fig. 6a,b) and increased retention of cargo in the lysosomal compartment over time (16 h) (Fig. 6c,d). These data suggest that progranulin-deficient macrophages take up apoptotic cells, but that lysosomal cargo processing is impaired. Progranulin has been reported to shuttle by its sortilin-binding domain to the lysosomal compartment^34^ and to regulate lysosomal acidification during autophagy^35^. As efficient lysosomal cargo destruction requires low pH in the lumen^36^, we measured lysosome acidification in WT and *GRN*^−/−^ macrophages during efferocytosis. In WT, but not in progranulin-deficient macrophages (*Grn* KO), the pH rapidly lowered and lysosomal acidification increased in phagolysosomes in response to efferocytosis (Fig. 6e,f). In progranulin-deficient cells (*GRN* KO), restoration of lysosomal acidification to WT level was seen on exogenous expression of recombinant full length progranulin, but not truncated progranulin lacking the sortilin-binding domain or isolated sortilin-binding domain (Extended Data Fig. 7a,b).

Upon proteolytic degradation of apoptotic cell cargo within the lysosome, lipid products are sensed by nuclear sterol receptors such as LXRα, which has previously been reported to regulate *Arg1* expression^37^. Pharmacological inhibition of LXRα (LXRi; GSK-2033) ablated efferocytosis-induced *Arg1* expression in efferocytic WT macrophages but had no additional effect on *Grn* KO macrophages (Fig. 6g). Furthermore, LXRα target gene, *Abca1* (ref. ^38^), was upregulated in WT, but not granulin deficient, efferocytic macrophages (Fig. 6h), suggesting reduced LXRα activity in efferocytic *Grn* KO macrophages. In WT, but not in *GRN* KO macrophages, efferocytosis markedly increased the interaction of LXRα (encoded by *Nr1h3*) with its heterodimer partner RXRα^38^ (Fig. 6i,j), whereas *Nr1h3* expression levels remained unchanged (Extended Data Fig. 7c). In agreement with increased LXRα activation in efferocytic WT macrophages, pharmacological blockade of LXRα reduced liver metastasis in vivo (Extended Data Fig. 7d,e).

Impairment of efferocytosis signaling has been associated with an antitumor type I interferon immune response^39^. We found that depletion of progranulin significantly increased activation of the type I interferon response, as evidenced by elevated levels of nuclear IRF3 (Extended Data Fig. 7f) and upregulation of *Ifnb1* expression (Extended Data Fig. 7g). Our data suggest that impaired lysosomal function in progranulin-deficient macrophages pauses LXRα-mediated *Arg1* expression and leads to type I interferon activation in response to impaired efferocytosis.

### Progranulin regulates lysosomal acidification via CFTR

**Fig. 7:**
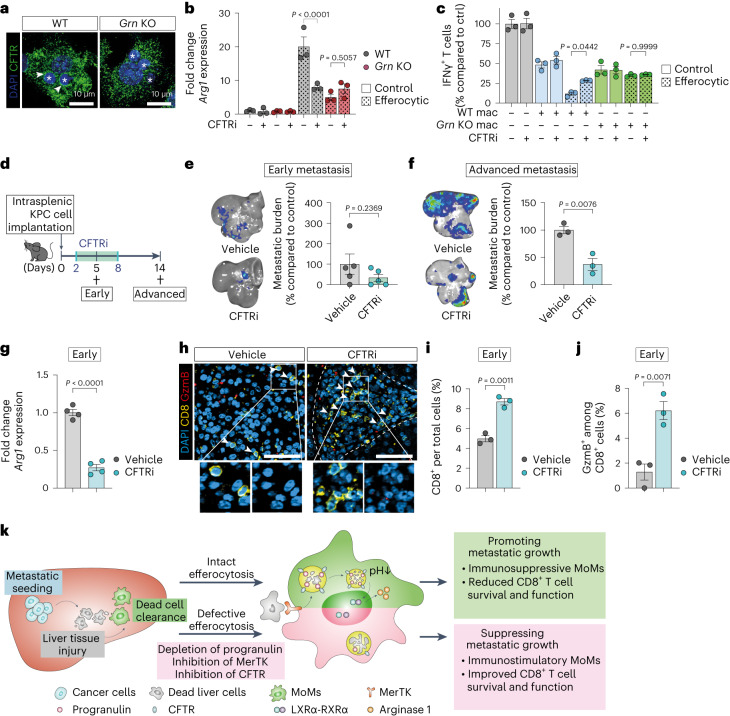
Inhibition of progranulin-regulated CFTR impairs efferocytosis-induced macrophage polarization. **a**, Representative immunofluorescence images of CFTR in efferocytic BMMs (experiment was performed twice with similar results). Asterisks indicate engulfed apoptotic thymocytes. **b**, qPCR analysis of *Arg1* in efferocytic BMMs (20 h). Error bars, mean ± s.e.m. (*n* = 3 biological replicates/group). *P* values, two-way ANOVA with Sidak’s post-test. **c**, Relative activation level of CD8^+^ T cells, measured as percentages of IFNγ^+^ cells, stimulated with anti-CD3/CD28-coupled Dynabeads and co-cultured with BMMs compared to Dynabead-only control (*n* = 3 biological replicates per group from one experiment). Error bars, mean ± s.e.m. *P* values, one-way ANOVA with Sidak’s post-test. **d**, Schematic illustrating the CFTRi experiment timeline. **e**,**f**, Representative ex vivo BLI images (left) and relative tumor burden (right) of d5 (*n* = 5 mice per group, from one experiment) (**e**) and d14 (**f**) livers (*n* = 5 mice per group, from two experiments). Error bars, mean ± s.e.m. *P* values, two-tailed unpaired t-test. **g**, qPCR analysis of *Arg1* in MoMs (F4/80^+^TIM4^−^) from d5 livers (*n* = 4 mice per group). Error bars, mean ± s.e.m. *P* value, two-tailed unpaired *t*-test. **h**–**j**, Representative immunofluorescence images (**h**) and quantification of total (**i**) and cytotoxic GzmB^+^ (**j**) CD8^+^ T cells in d5 livers (*n* = 3 mice per group, from one experiment). Arrowheads indicate CD8^+^ T cell. Scale bars, 50 µm. Error bars, mean ± s.e.m. *P* value, two-tailed unpaired *t*-test. **k**, Graphical schematic summarizing the role of efferocytic macrophages in PDAC liver metastasis. During early stage of metastasis, seeding of cancer cells induces liver injury leading to clearance of dead cell debris by monocyte-derived macrophages (MoMs) via receptor MerTK. Engulfed dead cells are degraded in acidic phagolysosome lumen, a process that is dependent on lysosomal acidification by progranulin (PGRN) and CFTR. Following efficient lysosomal degradation of the dead cell cargo, LXRα is activated and induces expression of the T cell inhibitory factor, arginase 1. Arginase 1-mediated reduction in T cell numbers and activation eventually facilitates metastatic outgrowth. Impairment in these processes and suppression of tumor growth can be achieved via depletion of progranulin or blockade of MerTK or CFTR functions. Source data

**Extended Data Fig. 8:**
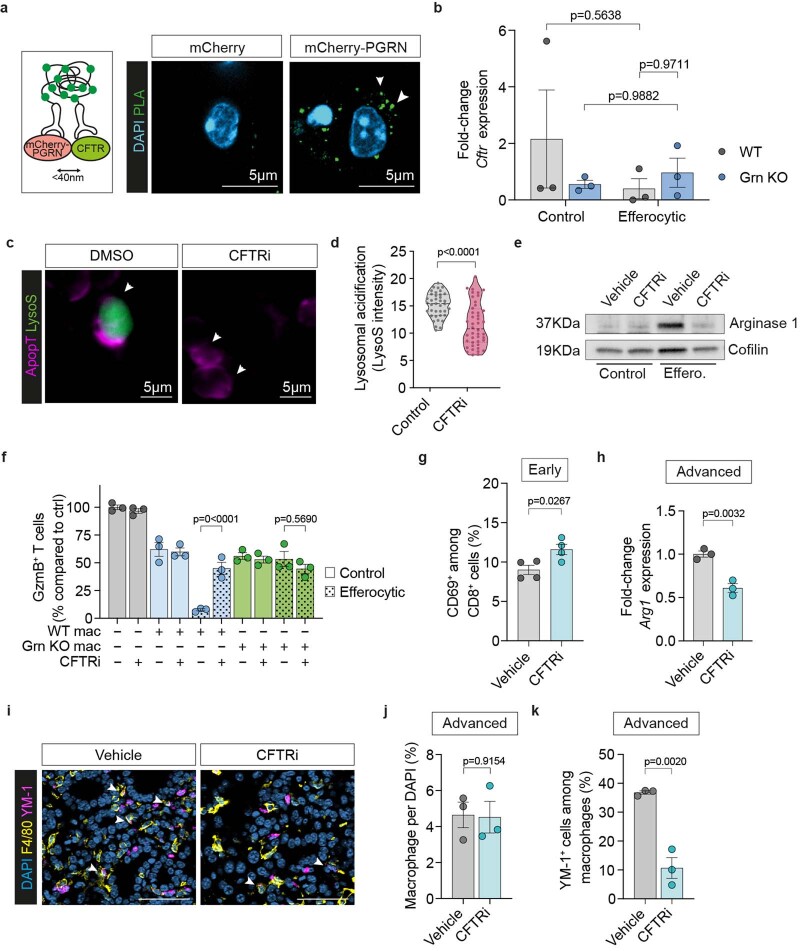
CFTR blockade impairs efferocytosis-induced macrophage conversion. **(a)** Schematic of proximity ligation assay (PLA) for mCherry and CFTR (left) and representative fluorescence images (right) of PLA probe-bound fluorophore (PLA, green) in BMMs transduced with mCherry-tagged PGRN (experiment was performed three times with similar results). **(b)** qPCR analysis of *Cftr* in BMMs (20hrs). Error bars, mean ± SEM (n = 3 biological replicates/group). P-values, two-way ANOVA with Sidak’s post-test. **(c)** Representative fluorescence images of LysoSensor-labeled BMMs (n = 38-44 cells/group, experiment was performed twice with similar results) incubated with CellTrace-labeled apoptotic thymocytes (ApopT). **(d)** Quantification of LysoSensor intensity as depicted in **(c)**. Error bars, mean ± SEM from one experiment. P-value, two-tailed unpaired t-test. **(e)** Representative Western blot image of Arginase 1 and loading control Cofilin levels in BMMs (3+16hrs, experiment was performed three times with similar results). **(f)** Relative activation level of CD8 + T cells, measured as percentages of GzmB+ cells, stimulated with anti-CD3/CD28-coupled Dynabeads and co-cultured with BMMs compared to Dynabeads-only control (n = 3 biological replicates/group from one experiment). Error bars, mean ± SEM. P-values, one-way ANOVA with Sidak’s post-test. **(g)** Flow cytometry analysis of activated (CD69+) CD8 + T-cells in d5 livers (n = 4 mice/group). Error bars, mean ± SEM. P-value, two-tailed unpaired t-test. **(h)** qPCR analysis of Arg1 in MoMs (F4/80 + TIM4–) from d14 livers (n = 3 mice/group). Error bars, mean ± SEM. P-value, two-tailed unpaired t-test. **(i-k)** Representative immunofluorescence images (i) and quantification of total (j) and YM-1+ (k) macrophages (F4/80+) in d14 livers. Arrowheads indicate YM-1+ macrophages (n = 3 mice/group, from one experiment). Scale bars, 50 µm. Error bars, mean ± SEM. P-value, two-tailed unpaired t-test. Source data

To identify the molecular mechanism by which progranulin regulates efferocytosis-induced lysosome acidification we analyzed potential binding partners listed in the interactome database IntAct. These included the cystic fibrosis transmembrane conductance regulator (CFTR), which regulates lysosome acidification in macrophages following phagocytosis of bacteria^40,41^. A proximity ligation assay of progranulin and CFTR confirmed their colocalization in the cytoplasm of WT macrophages (Extended Data Fig. 8a). Consistently, a lack of progranulin depletes the level of CFTR in lysosomes (Fig. 7a), whereas its expression levels remained unaffected (Extended Data Fig. 8b). Pharmacological inhibition of CFTR using CFTRinh172 (CFTRi) significantly impaired efferocytosis-induced lysosomal acidification in macrophages (Extended Data Fig. 8c,d). In addition, in vitro CFTR inhibition significantly reduced *Arg1* expression (Fig. 7b and Extended Data Fig. 8e) and ablated the increased immunosuppressive activities of efferocytic WT macrophages, but did not show any effect on efferocytic progranulin-deficient macrophages (Fig. 7c and Extended Data Fig. 8f).

To assess the impact of CFTR inhibition on PDAC liver metastasis, we performed intrasplenic implantation of KPC-derived cancer cells followed by treatment with CFTRi or vehicle control for seven consecutive days (Fig. 7d). CFTR inhibition caused significant reduction in metastatic tumor burden at advanced stage (Fig. 7e,f). In agreement with our in vitro finding, administration of CFTRi significantly reduced *Arg1* expression levels in MoMs (Fig. 7g), increased CD8^+^ T cell infiltration (Fig. 7h,i) and their activation (GzmB^+^CD8^+^; CD69^+^CD8^+^ T cells) in early metastatic tumors (Fig. 7i,j and Extended Data Fig. 8g).

In advanced metastasis, CFTRi-treated mice displayed sustained impaired *Arg1* expression in MoMs (Extended Data Fig. 8h) and inhibited macrophage conversion, demonstrated by overall reduced numbers of YM-1^+^ macrophages within metastatic lesions (Extended Data Fig. 8i–k). Together, our findings show that progranulin regulates lysosomal acidification in macrophages via CFTR and that pharmacological blockade of lysosomal acidification with a CFTR inhibitor inhibits PDAC liver metastasis growth (Fig. 7k).

## Discussion

Using a scRNA-seq and in situ cell-labeling approach, we reveal the spatial and temporal heterogeneity of macrophages in PDAC liver metastases. We confirm that macrophage tissue origin plays a key role in determining the localization and function of these cells in metastatic liver tumors. Recruited MoMs (TIM4^−^/VSIG4^−^) and tissue-resident KCs (TIM4^+^/VSIG4^+^) expand during metastatic disease progression; however, MoMs are located within metastatic tumor lesions, whereas KCs are mostly found at the periphery. Our finding is consistent with studies showing enrichment of resident macrophages at tumor-adjacent tissue^42–44^. Colonization of the liver by cancer cells induces KCs to display an inflammatory phenotype that is maintained throughout metastatic progression (cluster 1 and 4). At later stages of metastasis a subset of KCs acquires a pro-fibrotic signature (cluster 4), potentially contributing to hepatic stellate cell/fibroblast activation in advanced lesions and thereby helping to sustain the desmoplastic stroma in PDAC liver metastasis. In agreement with these findings, a pro-fibrotic phenotype of resident macrophages has recently been reported in primary PDAC and other cancers^45^. Our observed changes in KC clusters in response to metastatic tumors call for future studies to dissect the biological functions of these macrophages in PDAC liver metastasis. Considering MoM clusters, immunostimulatory and immunosuppressive phenotypes coexist in early and advanced metastatic lesions. Notably, phenotypic conversion toward an immunosuppressive subtype occurs in early and advanced stages of metastasis. Our data suggest that MoMs are constantly recruited to the site of metastasis where they rapidly lose their immunostimulatory potential.

In solid tumors, pan-macrophage targeted strategies using CSF-1 inhibitors as a single agent have shown very modest or no activity^13^. Pan-macrophage-targeted strategies will inevitably remove immunostimulatory and immunosuppressive macrophages. Our findings support future development of tailored approaches, targeting immunosuppressive macrophage clusters or regulators controlling macrophage conversion toward an immunosuppressive subset.

Despite our increasing understanding of macrophage heterogeneity, drivers of macrophage phenotypic and functional polarization in the metastatic microenvironment are not fully elucidated. The presence of dead cells in the tumor microenvironment can induce an immunosuppressive state in macrophages^7^. In normal physiology, efferocytosis-mediated clearance of debris and dead cells by macrophages is pivotal to mitigating inflammation during the resolution phase after injury and preventing chronic tissue damage^5,46^. Thus, efferocytosis is physiologically used to protect host tissues from immune attack, resulting in local immunosuppression. Here, we show that metastatic cancer cells hijack these evolutionary conserved and hard-wired pathways to create a local immunosuppressive microenvironment in the liver, allowing disseminated cancer cells to escape immune detection and grow.

Tissue-resident macrophages are reportedly more phagocytic than bone marrow-derived macrophages^47^; our observation suggests that MoMs accumulate at necrotic sites and are able to phagocytose dead resident cells. Reversal of the efferocytosis-induced immunosuppressive state can be achieved by inhibiting phosphatidylserine receptor MerTK. Blockade of MerTK-mediated phagocytosis of dying cancer cells suppresses tumor growth in other cancers^29,48–50^. In one study, MerTK blockade impaired macrophage-mediated clearance of apoptotic cells, increasing the accumulation of dead cell bodies and resulting in the activation of the STING pathway in macrophages via cGAMP/ATP^29^. We observed STING pathway activation in the absence of MerTK inhibition, likely due to the high accumulation of dead cells during metastasis-induced liver injury. In agreement, liver injury induces the STING pathway under non-cancerous conditions^51^.

In addition to TAM receptors, such as MerTK, macrophages can bind apoptotic cells via other phosphatidylserine receptors, such as CD300 or CD36 (ref. ^5^). Given the many efferocytosis receptor variants, we examined whether targeting the downstream phagolysosomal pathway could suppress the efferocytosis-induced immunosuppressive state in macrophages. Of note, uptake of extracellular accumulation of lipids has recently been shown to induce an immunosuppressive phenotype in macrophages^52^. As engulfed lipids are processed through the lysosomal machinery, impairing lysosomal function may also inhibit lipid-induced macrophage conversion, thereby representing an emerging checkpoint of macrophage functions.

Progranulin is an immunoregulatory lysosomal protein^53^. We have shown that progranulin has pro-tumorigenic effects and activates fibroblasts in advanced PDAC liver metastasis^9^. In this study, depletion of progranulin in efferocytic macrophages was sufficient to block upregulation of *Arg1* and restore T cell activation. Mechanistically, progranulin regulates the lysosomal acidification, via CFTR, which is required for optimal processing of apoptotic cell cargo and subsequent LXRα-mediated upregulation of *Arg1* (Fig. 7m). Consequently, CFTR or LXRα inhibition also result in macrophage conversion and T cell stimulation. Further studies will be needed to explore whether targeting efferocytosis (MerTK), lysosomal degradation (progranulin/CFTR) or downstream LXRα activation will be beneficial, in combination with hepatotoxic chemotherapy or immune-checkpoint therapies, to harness increased antitumor immune responses.

In summary, our findings showed that PDAC liver metastasis induces macrophage heterogeneity, that metastasis-promoting and restricting subclusters coexist in metastatic livers, and that interfering with macrophage efferocytosis, or its downstream signaling events, inhibits macrophage immunosuppressive functions and restores antitumor immunity. Targeting macrophage efferocytosis may be an attractive new treatment strategy for patients with metastatic PDAC.

## Methods

### Ethics statement

This study complies with all relevant ethical regulations. Studies involving the use of liver metastasis biopsy and blood samples from patients with treatment-naive, advanced PDAC were accessed using the PINCER platform study, approved by the National Research Ethics Service Committee North West, Greater Manchester REC15/NW/0477. All individuals provided written informed consents on approved institutional protocol. All animal studies were conducted in accordance with UK Home Office regulations under project license P16F36770.

### Mice

Mice were housed under specific-pathogen-free conditions at the Biomedical Science Unit at the University of Liverpool. C57BL/6 mice were obtained from Charles River Laboratories. *Grn*^−/−^ (B6(Cg)-*Grn*^tm1.1Aidi^), *Grn*^fl/fl^ (C57BL/6-*Grn*^tm1Aidi^) and tdTomato^+^ mice (B6.129(Cg)–Gt(ROSA)26Sor^tm4(ACTB–tdTomato,–EGFP)Luo^) all on a C57BL/6 genetic background were purchased from The Jackson Laboratory. Tamoxifen-inducible *Csf1r-Cre* mice (BL6-Tg(Csf1r-cre/Esr1*)1Jwp/J) on a C57BL/6 background were kindly provided by J.W. Pollard’s laboratory (University of Edinburgh). For animal studies, female mice aged 6–8 weeks old were used, except for the *Grn* KO study in spontaneous liver metastasis model, which used male mice. The maximum tumor burden limit of 1.5 cm mean diameter was not exceeded in the studies. In all animal studies, the severity was limited to moderate.

### Autochthonous KPC model

KPC (*Kras*^G12D/+^; *Trp53*^R172H^; *Pdx1-Cre*) mice on mixed^54^ and pure C57BL/6 backgrounds were bred in-house at the Cancer Research UK (CRUK) Beatson Institute and maintained with environmental enrichment, access to standard chow and water ad libitum. All animal experiments were performed under a UK Home Office license and approved by the University of Glasgow Animal Welfare and Ethical Review Board. Genotyping was performed by Transnetyx. Tissues were collected after primary tumor development or at a humane time point.

### Cells

The murine pancreatic cancer cell line KPC FC1199 (kindly provided by the Tuveson Laboratory, Cold Spring Harbor Laboratory) was isolated from PDAC tumor tissues of KPC (*Kras*^G12D/+^; *Trp53*^R172H^; *Pdx1-Cre*) mice on a pure C57BL/6 background and authenticated as previously reported^55^. KPC^Luc/ZsGreen^ cells were generated using pHIV Luc-ZsGreen (a gift from B. Welm, University of Utah; Addgene plasmid 39196) through lentivirus infection. Infected cells were selected for high ZsGreen expression levels using a FACSAria III cell sorter (BD Biosciences). Human cell lines HEK293T (CRL-3216), THP-1 (TIB-202) and Jurkat (TIB-152) were obtained from ATCC. THP-1 cells were incubated with 50 nM phorbol 12- myristate 13-acetate (PMA) for 72 h to generate THP-1-derived macrophages.

All cell lines were maintained in RPMI (for THP-1 and Jurkat) or DMEM (for others) supplemented with 10% FBS and antibiotic antimycotic solution (10 U ml^−1^ penicillin, 0.1 mg ml^−1^ streptomycin and 0.25 µg ml^−1^ amphotericin B) (Sigma) and tested negative for *Mycoplasma*. The cell lines used in this article are not listed in the International Cell Line Authentication Committee and National Center for Biotechnology Information Biosample database of misidentified cell lines.

Primary murine BMMs were generated by flushing BM from femurs and tibias followed by incubation with 10 ng ml^−1^ murine M-CSF (Peprotech) for 5 d. Primary human MoMs were generated by isolating peripheral blood mononuclear cells (PBMCs) using gradient centrifugation, followed by incubation with 50 ng ml^−1^ human M-CSF (Peprotech) for 7 d.

### Mouse organoid isolation and culture

Metastatic liver organoid cells were isolated from KPC (*Kras*^G12D/+^; *Trp53*^R172H^; *Pdx1-Cre)* mice on a C57BL/6 background with advanced liver metastasis using a previously described method^56^. Briefly, metastatic liver tissues were minced and digested at 37 °C using 0.125 mg ml^−1^ Collagenase Crude Type XI and 0.125 mg ml^−1^ Dispase II for 2–4 h, followed by further digestion using TrypLE and at 37 °C for 10 min. Afterwards, digested cells were seeded in Growth Factor-Reduced Matrigel (Corning) and cultured in Advanced DMEM/F12 feeding medium (Thermo Fisher) containing 0.5 μM A83-01 (Tocris), 0.05 μg ml^−1^ mEGF (Thermo Fisher), 0.1 μg ml^−1^ FGF-10 (Peprotech), 0.01 μM Gastrin I (Tocris), 0.1 μg ml^−1^ mNoggin (Peprotech), 1.25 mM *N*-acetylcysteine (Sigma), 10 mM nicotinamide (Sigma), 1× R-Spondin-containing medium and 1× B27 supplement (Thermo Fisher). When organoids were first isolated, thawed or dissociated into single cells, 10.5 mM Y-27632 was added for the first passage.

### Liver metastasis models

Experimental liver metastasis was induced by injecting 1 × 10^6^ KPC^Luc/ZsGreen^ in PBS into the spleen. For the scRNA-seq study, primary tumor formation was induced by pancreatic injection of 2 × 10^5^ KPC^Luc/ZsGreen^ in Matrigel. Ten days after implantation, when primary tumors reached 200–250 mm^3^, liver metastasis was induced by injecting 1 × 10^6^ KPC^Luc/ZsGreen^ in PBS into the portal vein. Mice were killed at the indicated time points and metastatic tumor burden in the liver was measured as total flux by ex vivo bioluminescence imaging using IVIS spectrum imaging system (PerkinElmer).

Spontaneous liver metastasis was induced by implanting 1 × 10^5^ metastatic PDAC liver organoid cells in Matrigel into the pancreas.

### BM transplantation

Recipient tdTomato^+^ mice were given 10 Gy irradiation, followed by tail vein injection of 5 × 10^6^ donor C57BL/6 BM cells. Approximately 4 weeks after transplantation, BM reconstitution was assessed by flow cytometry of peripheral blood cells. Mice with a chimerism level of >50% were intrasplenically injected with KPC cells to induce liver metastasis. At day 5 after implantation, livers were collected for analysis.

### TCM

To prepare the TCM, KPC cells were grown to 70–80% confluence, after which the medium was discarded and replaced with low-serum (2% FBS) medium. After 24 h, the medium was collected and injected at 10 ml kg^−1^ to the tail vein of C57BL/6 mice.

### Drug treatments

MerTK inhibitor UNC2250 (APExBIO) was administered at 10 mg kg^−1^ dose via oral gavage once daily in experimental metastasis studies and once every 2 d in the spontaneous metastasis study. For the CD8^+^ T cell depletion study, 100 µg of anti-mouse CD8α (clone 2.43, BioXcell) or IgG2b isotype control (BioXcell) was administered via intraperitoneal injection every 3 d. For induction of liver injury, a single dose (100 mg kg^−1^) of *N*-acetyl-para-aminophenol (APAP, Sigma) was administered via intraperitoneal injection 2 d before induction of liver metastasis. For *Csf1r-Cre*^+^; *Grnf*^f/f^ and *Csf1r-Cre*^−^;*Grn*^f/f^ studies, tamoxifen was administered via oral gavage at 100 mg kg^−1^ dose once daily in experimental metastasis model and once every 2 d in spontaneous metastasis model. LXRα inhibitor GSK-2033 (Axon Medchem) was given once daily via intraperitoneal injection at a dose of 10 mg kg^−1^. CFTR inhibitor CFTRinh172 (Selleckchem) was administered via intraperitoneal injection at a dose of 1.25 mg kg^−1^ twice a day.

### Liver cell isolation

Single-cell suspensions from murine livers were prepared by mechanical and enzymatic disruption with 1 mg ml^−1^ Collagenase P (Roche) in Hanks balanced salt solution at 37 °C for 30–40 min. Cells were then incubated with 0.05% trypsin at 37 °C for 5 min. After removal of debris by filtering the cell suspension through a 70-µm strainer, red blood cells were removed using RBC Lysis Buffer (BioLegend).

### Flow cytometry and cell sorting

Liver single-cell suspensions were prepared as above, followed by resuspension in MACS buffer (0.5% BSA, 2 mM EDTA and PBS) and incubation with anti-mouse CD16/CD32 (BD Biosciences) for 10 min on ice. For cell surface staining, cells were incubated with SYTOX Blue viability marker (Thermo Fisher) and fluorophore-conjugated antibodies (BioLegend; Supplementary Table 4).

For the T cell activation assay, following Fc receptor blocking, cells were incubated with a LIVE/DEAD Fixable Aqua Dead Cell Stain kit (Thermo Fisher) and fluorophore-conjugated CD8 antibody (BioLegend). Cells were then fixed using IC Fixation Buffer and permeabilized using Intracellular Staining Perm Wash Buffer (BioLegend) according to the manufacturer’s instructions, followed by staining with fluorophore-conjugated IFNγ and granzyme B antibodies (BioLegend; Supplementary Table 4). For apoptotic T cell measurement, cells were incubated with Apotracker Green (BioLegend) for 20 min and co-stained with CD8 antibody (BioLegend).

Flow cytometry data were acquired on a FACS Canto II (BD Biosciences) using FACSDiva (BD Biosciences) software and analyzed using FlowJo v.10 software (gating strategies in Supplementary Figs. 1–6). Cell sorting was performed on a FACS Aria III cell sorter (BD Biosciences). Macrophages were sorted into RLT buffer + β-mercaptoethanol according to the manufacturer’s instruction for RNA isolation (QIAGEN) or in DMEM supplemented with 20% FBS for the T cell activation assay.

### Efferocytosis assay

Before assay, primary murine BMMs or human MoMs were cultured in M-CSF-free and THP-1 cells in PMA-free media for 24 h. Apoptosis in primary murine thymocytes, human lymphocytes or Jurkat cells was induced with 1 µM staurosporine for 24 h. Apoptotic cells were added to macrophages at a 10:1 (apoptotic cells:macrophages) ratio. In some instances, drugs (10 µM UNC2250/5 µM GSK-2033/10 µM STING inhibitor H-151 (Tocris)/30 µM CFTRinh-172) were added to BMMs 30 min before incubation with apoptotic thymocytes. Macrophages were incubated with apoptotic cells either for 3 h, followed by a washout and further 16-h incubation (3 + 16 h) or for 20 h unless otherwise stated. For the T cell activation assay, before co-culture with CD8^+^ T cells, BMMs were co-cultured with apoptotic thymocytes for 4 h and then washed.

### T cell activation assay

Primary murine splenocytes were activated with Dynabead-conjugated CD3/CD28 at a 2:1 (splenocytes:beads) ratio and co-cultured with BMMs at a 20:1 (splenocytes:BMMs) ratio for 24 h. Cells were then incubated with 1× Brefeldin A Solution (BioLegend) for 4–6 h before immunostaining and flow cytometry analysis as described above.

### Phagosomal cargo degradation

Apoptotic thymocytes were stained with Vybrant DiD Cell-Labeling Solution (Thermo Fisher) following the manufacturer’s protocol. BMMs were incubated for 3 h with apoptotic thymocytes followed by thorough washing. Cells were then either fixed for 10 min at room temperature with 10% formalin (0 h) or left for further 16 h to allow for cargo degradation and fixed the next day. Cells were imaged using Axio Observer Z1 microscope (Zeiss).

### Phagolysosomal acidification assay

Lysosomal acidification assay was performed using the IncuCyte pHrodo Red Cell-Labeling kit (Sartorius) or LysoSensor DND-189 (Thermo Fisher). For assay using pHrodo, apoptotic thymocytes were stained with 1 µg ml^−1^ of pHrodo at 37 °C for 1 h and then with 2.5 µM CellTracker CFSE (Thermo Fisher) at 37 °C for 20 min.

For assays using LysoSensor, 2 h before the assay, BMMs were stained with 1 µM LysoSensor Green DND-189 (Thermo Fisher) following the manufacturer’s protocol. Apoptotic thymocytes were stained with 5 µl ml^−1^ Vybrant DiD for 45 min at 37 °C in the dark and incubated with BMMs.

Live-cell imaging was performed on a Cell Discoverer 7 or LSM800 microscopes (Zeiss). Peak intensity of pHrodo or LysoSensor was analyzed using Zen v.3.8 (Zeiss) or CellTracker (Warwick University).

### PLA

A PLA was performed using the Duolink PLA kit (Sigma) according to the manufacturer’s guidelines. Briefly, following isolation and differentiation for 5 d, primary murine BMMs were seeded onto coverslips and left overnight to adhere. BMMs were then fixed with 4% paraformaldehyde and permeabilized with 0.025% Triton-X/PBS, followed by incubation with LXRα and RXRα antibodies (Supplementary Table 4) overnight at 4 °C. BMMs were then incubated with PLA probes, followed by ligation and amplification steps. Image data were acquired on an LSM800 microscope (Zeiss) and analyzed using Zen v.3.8 software (Zeiss).

### Immunohistochemistry

Deparaffinization and antigen retrieval were carried out using the PT-Link System (Dako), followed by immunostaining using the EnVision Plus System (Dako). Tissue sections were incubated with a primary antibody (Supplementary Table 4) at 4 °C overnight, followed by incubation with an HRP-conjugated polymer. Staining was developed using diaminobenzidine and counterstained with hematoxylin (Sigma). Image data were acquired on an Axio Observer Z1 microscope (Zeiss) and analyzed using Zen v.3.8 software (Zeiss).

### Immunofluorescence

For tissue staining, deparaffinization and antigen retrieval was carried out using the PT-Link System (Dako), followed by blocking using 10% normal goat serum. Tissue sections were then incubated with primary antibodies (Supplementary Table 4) at 4 °C overnight, followed by incubation with fluorophore-conjugated secondary antibodies (Supplementary Table 4) and nuclear dye DAPI (Thermo Fisher) at room temperature for 1 h. Tissue sections were then mounted using Fluorescent Mounting Medium (Dako). For staining using two antibodies raised from the same host species, tyramide signal amplification was performed using a Tyramide Superboost kit (Thermo Fisher) according to the manufacturer’s guidelines. Image data were acquired on an Axio Observer Z1 microscope (Zeiss) and analyzed using Zen v.3.8 software (Zeiss).

For immunofluorescence staining of primary murine BMMs, cells were cultured in µ-Plate 24-well black (ibidi) and fixed with 4% paraformaldehyde in PBS, pH 7.4, for 10 min at room temperature. Cells were permeabilized for 10 min in Permeabilization Buffer (eBioscience) containing 0.1% saponin and blocked for 1 h in 10% donkey serum and 0.1% saponin buffer. Cells were then incubated with primary antibodies (Supplementary Table 4) overnight at 4 °C, followed by incubation with fluorophore-conjugated secondary antibodies (Supplementary Table 4) and nuclear dye DAPI for 2 h at room temperature. Image data were acquired on an LSM800 microscope (Zeiss) and analyzed using Zen v.3.8 software (Zeiss).

### RT–qPCR

RNA was extracted from primary murine BMMs or isolated liver MoMs using RNeasy kit (QIAGEN) and cDNA was synthesized using Quantitect Reverse Transcription kit (QIAGEN). Real time PCR (Supplementary Table 3) was performed using EvaGreen reagent (Solis Biodyne) according to the manufacturer’s guideline on AriaMX Real Time PCR System (Agilent).

### Immunoblotting

Protein lysates were prepared using a RIPA Lysis Buffer System (Santa Cruz). Protein concentration was determined using a Pierce Protein BCA Assay kit (Thermo Fisher) according to the manufacturer’s guidelines. Equal amounts of proteins were separated on TGX Precast Gel (Bio-Rad) and blotted using a Trans-blot Turbo Transfer System (Bio-Rad). Membranes were blocked in 5% BSA in Tris-buffered saline containing 0.1% Tween-20 (TBST), followed by incubation with primary antibodies (Supplementary Table 4) at 4 °C overnight. After washing in TBST, membranes were incubated with HRP-conjugated secondary antibodies (Supplementary Table 4). The protein bands were visualized using the Pierce ECL Western Blotting Substrate (Thermo Fisher) and imaged using Chemidoc Imaging System (Bio-Rad).

### Cloning

mCherry and mGrn inserts generated using mCherry–mPGRN-F1 and mCherry–mPGRN-F2 primer pairs (Supplementary Table 3), respectively, were cloned into pHIV vector plasmid using SLiCE method^57^ by incubating 1 µl 10× SLiCE buffer, 1 µl SLiCE extract, 50 ng linearized vector DNA and DNA inserts at a molar ratio of 10:1 for 1 h at 37 °C. Insertions were confirmed by sequencing using ef1.F and mcherry.R or mPGRNclonecheck.F and pHIV.rev primers (Supplementary Table 3).

To generate GRN KO HEK293T cells, a pair of guides (CCCTTGTGCCCTCATTCATG and GACTGAGTGACCCTAGAATCA) were used to delete approximately 1 kb of DNA from exon 2 to exon 4 of GRN. Complementary guide RNA (sgRNA) sequence primers, guide 1 FP/RP and guide 2 FP/RP (Supplementary Table 3), were phosphorylated using T4 Polynucleotide kinase (Thermo Fisher) following the manufacturer’s protocol. Phosphorylated products were ligated into LentiCRISPRv2 at 4 °C overnight using T4 DNA Ligase (Thermo Fisher). PCR with hU6-F and guide-specific RP primers were used for sequence verification.

HEK293T cells were transfected with 1 µg sgRNA constructs using Lipofectamine 2000 (Thermo Fisher). The medium was changed 24 h later and puromycin (Thermo Fisher) was added for 48 h. Cells with successful double deletion were expanded and lack of PGRN expression was confirmed using immunoblotting.

### Bulk RNA sequencing

Liver biopsy tissues from patients with pathologically confirmed liver metastasis were homogenized using a Minilys homogenizer (Bertin). Total RNA was extracted using RNAeasy kit (QIAGEN) and genomic DNA was removed using TURBO DNA-free kit (Thermo Fisher). Sequencing libraries were prepared using the SMARTer Stranded Total RNA-Seq Kit v2, Pico Input Mammalian kit (Takara) and sequenced on an Illumina’s NextSeq500 instrument.

### scRNA-seq

In situ antibody labeling of liver macrophages in mice with advanced metastasis was performed using a retrograde perfusion of the liver as previously described^58^ with 10 µg FITC-conjugated F4/80 antibody. Single-cell suspensions from unlabeled (healthy and early metastasis) and antibody-labeled (advanced metastasis) livers, as well as pre-metastatic and organoid-induced metastatic livers, were prepared as outlined above and stained with SYTOX Blue (Thermo Fisher), CD45, CD11b and APC-conjugated F4/80 antibodies (Supplementary Table 4). Live macrophages (SYTOX^−^CD45^+^CD11b^+^F4/80^+^) were sorted using a FACSAria III cell sorter (BD Biosciences) and processed for library preparation using the 10x Genomics Chromium Chip B Single Cell kit and Single Cell 3′ GEM, Library & Gel Bead kit (10x Genomics), according to the manufacturer’s protocol. Paired-end sequencing was performed using an Illumina NovaSeq 6000 instrument.

FASTQ files were trimmed and filtered to remove low-quality reads. Reads were converted to expression matrices using CellRanger v.3.0.2 (10x Genomics) using the mouse reference genome version mm10 available from the 10x Genomics website. Data were loaded into R v.3.6.1 using the Seurat library, which was used for all subsequent analysis unless stated otherwise. Genes that were detected in fewer than 20 cells as well as mitochondrially-encoded and ribosomal subunit genes were removed. Cells for which fewer than 200 (low quality) or more than 6,000 (higher probability of duplets) genes were detected and removed, as were cells for which mitochondrially-encoded genes made up greater than 10% of the total expression and those that did not express either pan-macrophage marker *Cd68* or *Adgre1* mRNA. The final experimental model dataset contained 9,171 cells (2,428 healthy, 2,007 early, 1,953 proximal advanced and 2,783 distal advanced) and 13,005 genes. For analysis of MoM subpopulation, 2,820 cells from the experimental model dataset were included. The pre-metastatic and spontaneous model dataset contained 1,366 cells (294 pre-metastatic and 1,022 advanced metastasis).

Data were normalized using the ‘LogNormalize’ method and then scaled. Genes with more variation than could be explained by technical factors were detected using the ‘vst’ method (*n* = 2,000 genes). Principal-component analysis (PCA) was performed using the RunPCA function, followed by inspection of an elbow plot, which revealed that 20 principal components captured most of the variation in the data. UMAP projections were then calculated using the top 20 principal components. A nearest neighbor map between cells was calculated using the FindNeighbors function, using the top 20 principal components from the PCA to inform the distance matrix. Cell clusters were detected using the FindClusters function, which uses an implementation of the Louvain algorithm, with the resolution parameter set to 0.44. DEGs between clusters were detected using the Wilcoxon rank-sum test as implemented in the FindMarkers function. After Bonferroni correction, genes with an adjusted *P* value less than 0.01 were deemed significant unless stated otherwise.

### Pathway and signature enrichment analyses

Signature enrichment analysis in bulk RNA-seq data was performed using the R package GSVA, using the ssGSEA method and normalizing scores. The GO BP pathway enrichment analysis of DEGs was performed using g:Profiler.

### Statistics and reproducibility

No statistical methods were used to predetermine sample sizes, but we used adequate numbers of samples that would provide statistically significant results based on our previous experience with the tumor models. Animal studies were performed twice, with the exceptions of the MerTKi and *Grn* KO spontaneous liver metastasis studies, which were performed once. In vitro experiments were repeated three times unless otherwise specified. No data were excluded from the analyses. The experiments were not randomized; however, mice with comparable age and body weight were assigned into control and experimental groups. The exception of this was the MerTKi study in spontaneous liver metastasis model where, before drug treatment, mice with comparable primary tumor burden were randomly assigned. The investigators were not blinded to allocation during experiments and outcome assessment. Data distribution was assumed to be normal but this was not formally tested.

Statistical analysis was performed using GraphPad Prism v.8 software (GraphPad). A *P* value of <0.05 was considered statistically significant. In some graphs, *P* < 0.0001 was indicated, as GraphPad Prism does not provide the exact values of *P* when it was lower than 0.0001.

### Reporting summary

Further information on research design is available in the Nature Portfolio Reporting Summary linked to this article.

### Supplementary information

Supplementary Information Supplementary Figs. 1–6.

Reporting Summary

Supplementary Table 1 Supplementary Tables 1–4.

### Source data

Source Data Fig. 1 Statistical source data.

Source Data Fig. 2 Statistical source data.

Source Data Fig. 3 Statistical source data.

Source Data Fig. 4 Statistical source data.

Source Data Fig. 5 Statistical source data.

Source Data Fig. 6 Statistical source data.

Source Data Fig. 7 Statistical source data.

Source Data Extended Data Fig. 1 Statistical source data.

Source Data Extended Data Fig. 2 Statistical source data.

Source Data Extended Data Fig. 3 Statistical source data.

Source Data Extended Data Fig. 4 Statistical source data.

Source Data Extended Data Fig. 5 Statistical source data.

Source Data Extended Data Fig. 6 Statistical source data.

Source Data Extended Data Fig. 7 Statistical source data.

Source Data Extended Data Fig. 8 Statistical source data.

Source Data Figs. 3,5 and 7 and Extended Data Fig. 4 Unprocessed western blot images.

## Data Availability

Mouse scRNA-seq were submitted to the Gene Expression Omnibus repository and can be accessed under accession no. GSE215118. Human bulk RNA-seq data are available from the authors upon reasonable request and subsequent Data Transfer Agreement to protect patients’ privacy. All other data supporting the findings of this study are available from the corresponding author on reasonable request. Source data are provided with this paper.

## Code availability

All code used to analyze data in this study is available on GitHub at https://github.com/CBFLivUni/Astuti_et_al_2023.
