# Author Correction: Efferocytosis reprograms the tumor microenvironment to promote pancreatic cancer liver metastasis

**DOI:** 10.1038/s43018-024-00751-y

**Published:** 2024-03-12

**Authors:** Yuliana Astuti, Meirion Raymant, Valeria Quaranta, Kim Clarke, Maidinaimu Abudula, Olivia Smith, Gaia Bellomo, Vatshala Chandran-Gorner, Craig Nourse, Christopher Halloran, Paula Ghaneh, Daniel Palmer, Robert P. Jones, Fiona Campbell, Jeffrey W. Pollard, Jennifer P. Morton, Ainhoa Mielgo, Michael C. Schmid

**Affiliations:** 1 Department of Molecular and Clinical Cancer Medicine, University of Liverpool, Liverpool, UK; 2 Computational Biology Facility, University of Liverpool, Liverpool, UK; 3 Cancer Research UK Scotland Institute, Glasgow, UK; 4 School of Cancer Sciences, University of Glasgow, Glasgow, UK

**Keywords:** Cancer microenvironment, Metastasis, Tumour immunology, Cancer

Correction to: *Nature Cancer* 10.1038/s43018-024-00731-2, published online 14 February 2024.

In the version of this article initially published, the H&E images of Control and TCM-treated livers in Fig. 3b were mislabelled as “TCM” and “Control”, respectively. This has now been corrected in the HTML and PDF versions of the article.

