# Supplementary material for: Efferocytosis reprograms the tumor microenvironment to promote pancreatic cancer liver metastasis
Source: Nat Cancer. 2024 Feb 14;5(5):774–90. doi: 10.1038/s43018-024-00731-2 (PMC11136665; doi:10.1038/s43018-024-00731-2)
Supplement: Supplementary file 1 — Supplementary Figs. 1–6. [file 43018_2024_731_MOESM1_ESM.pdf]

nature cancer

Article https://doi.org/10.1038/s43018-024-00731-2

Efferocytosis reprograms the tumor microenvironment to promote pancreatic cancer liver metastasis

In the format provided by the authors and unedited

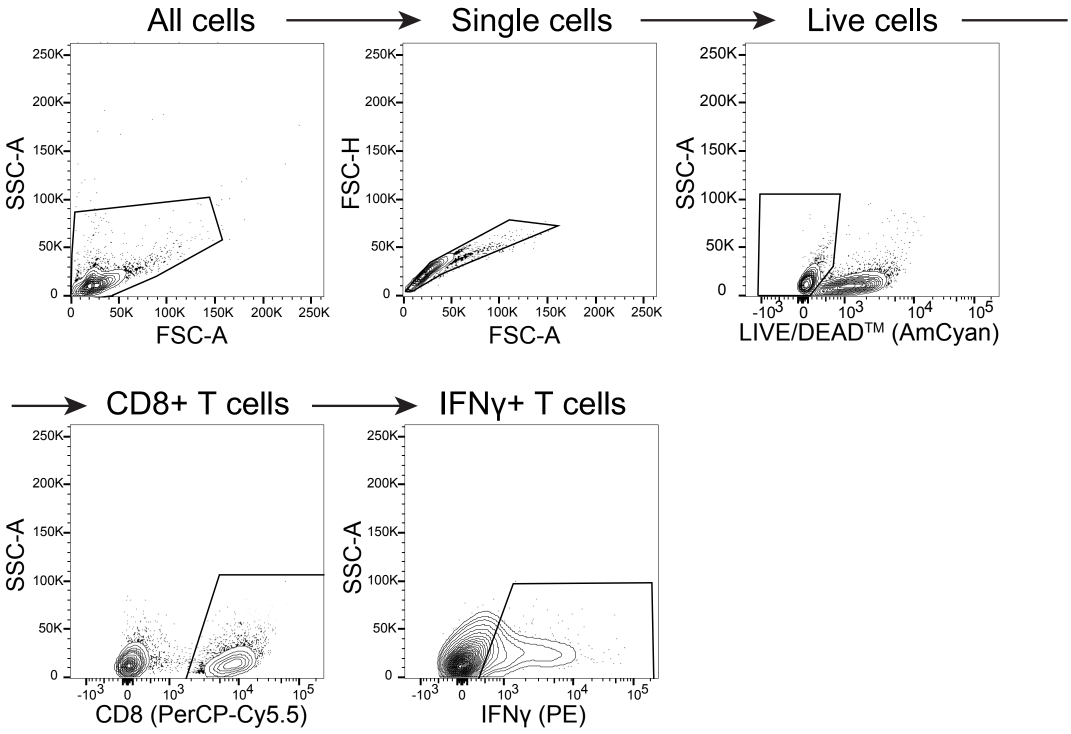

Supplementary Figure S1. FACS gating strategy of activated (IFNγ+) CD8+T cells.

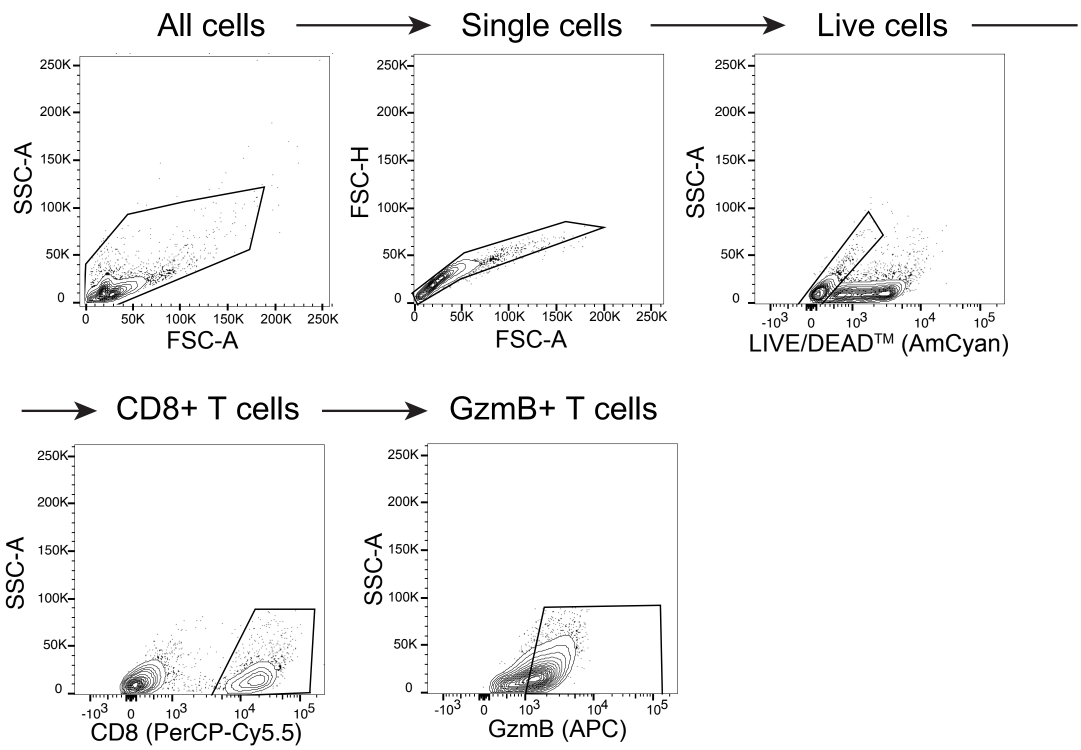

Supplementary Figure S2. FACS gating strategy of cytotoxic (GzmB+) CD8+ T cells.

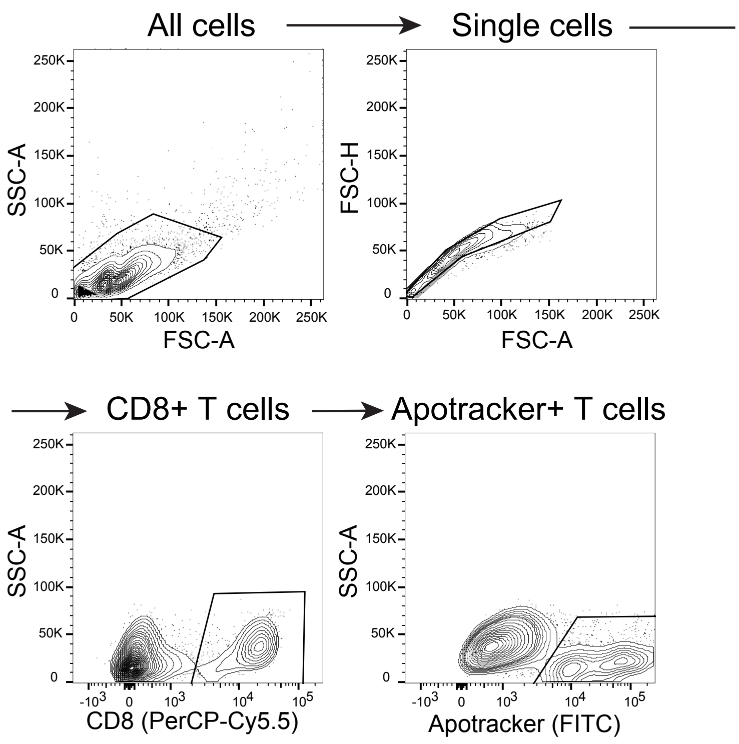

Supplementary Figure S3. FACS gating strategy of apoptotic (Apotracker+) CD8+ T cells.

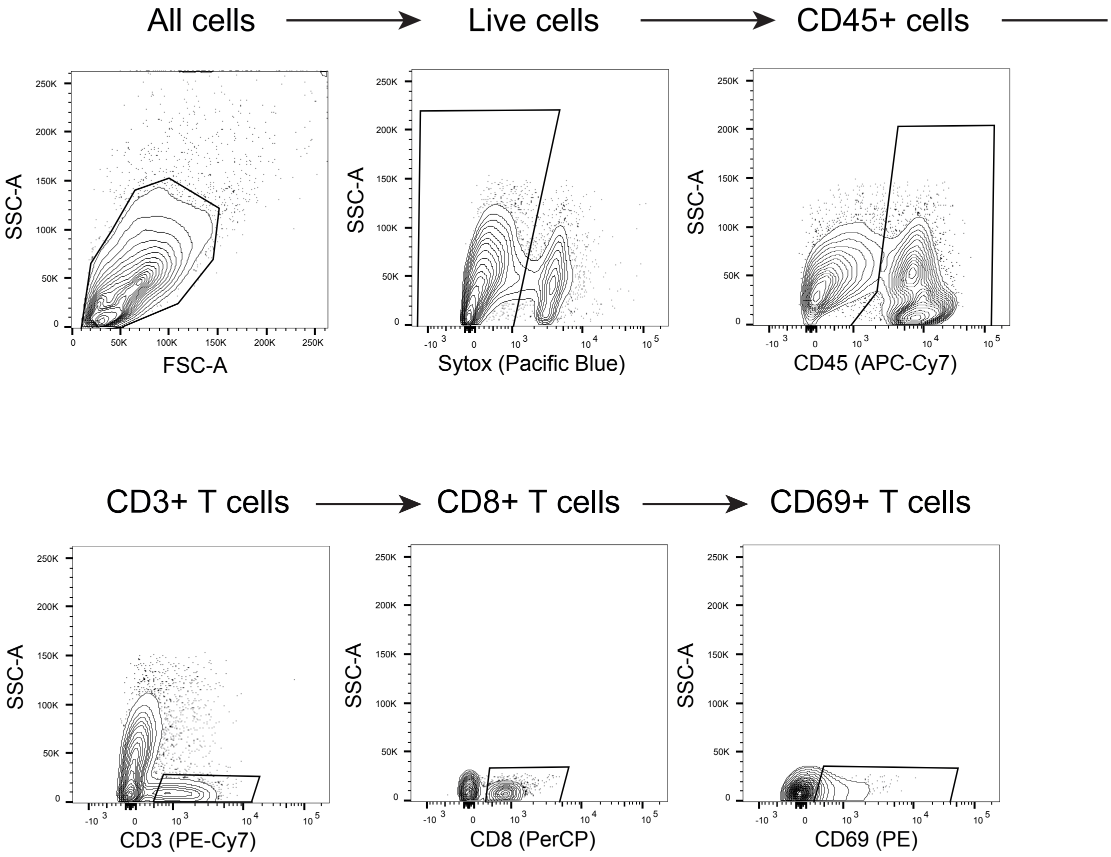

Supplementary Figure S4. FACS gating strategy of activated (CD69+) CD8+ T cells

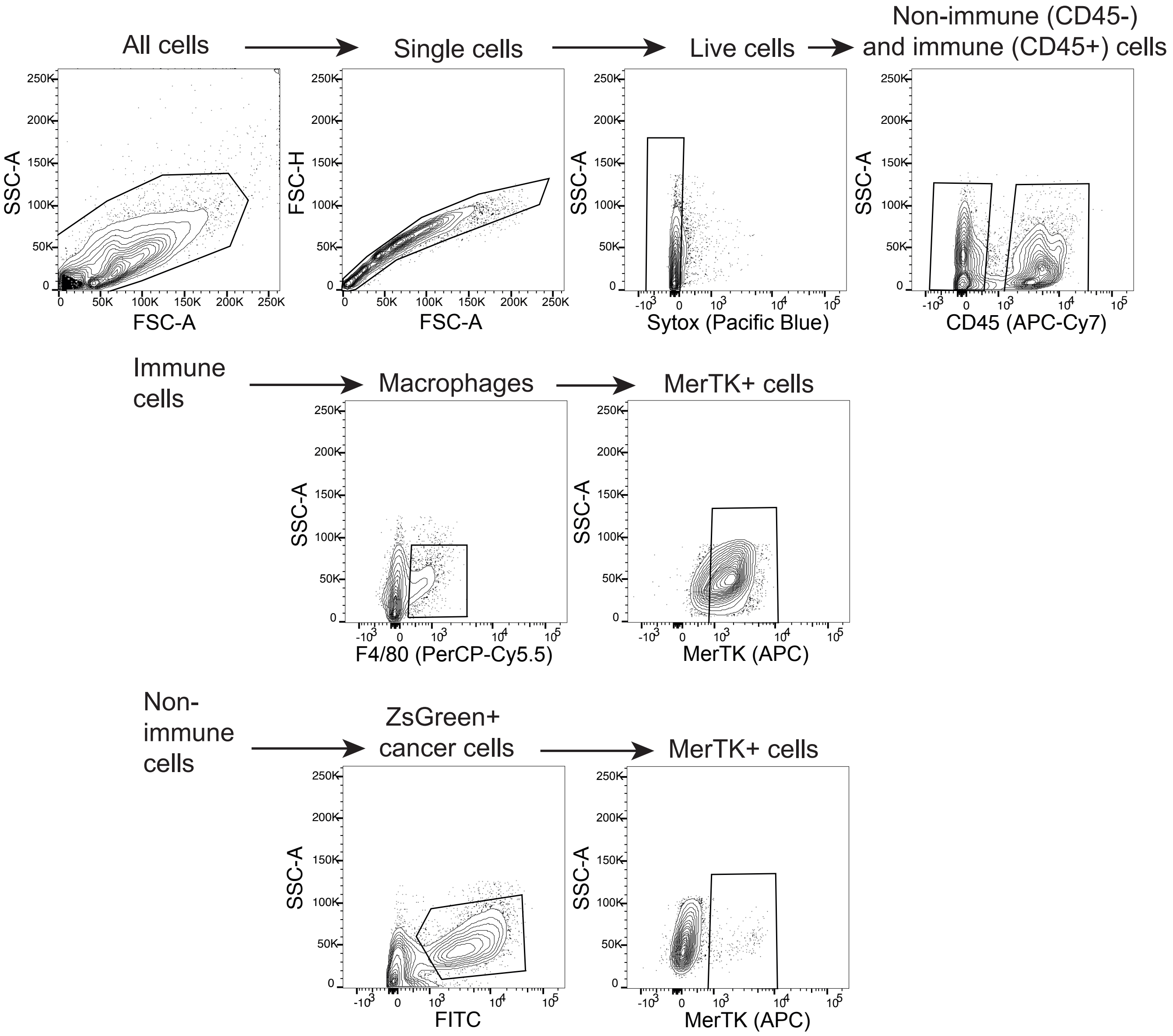

Supplementary Figure S5. FACS gating of MerTK+ cells in experimental liver metastasis using Luciferase+/ZsGreen+ FC1199 cells.

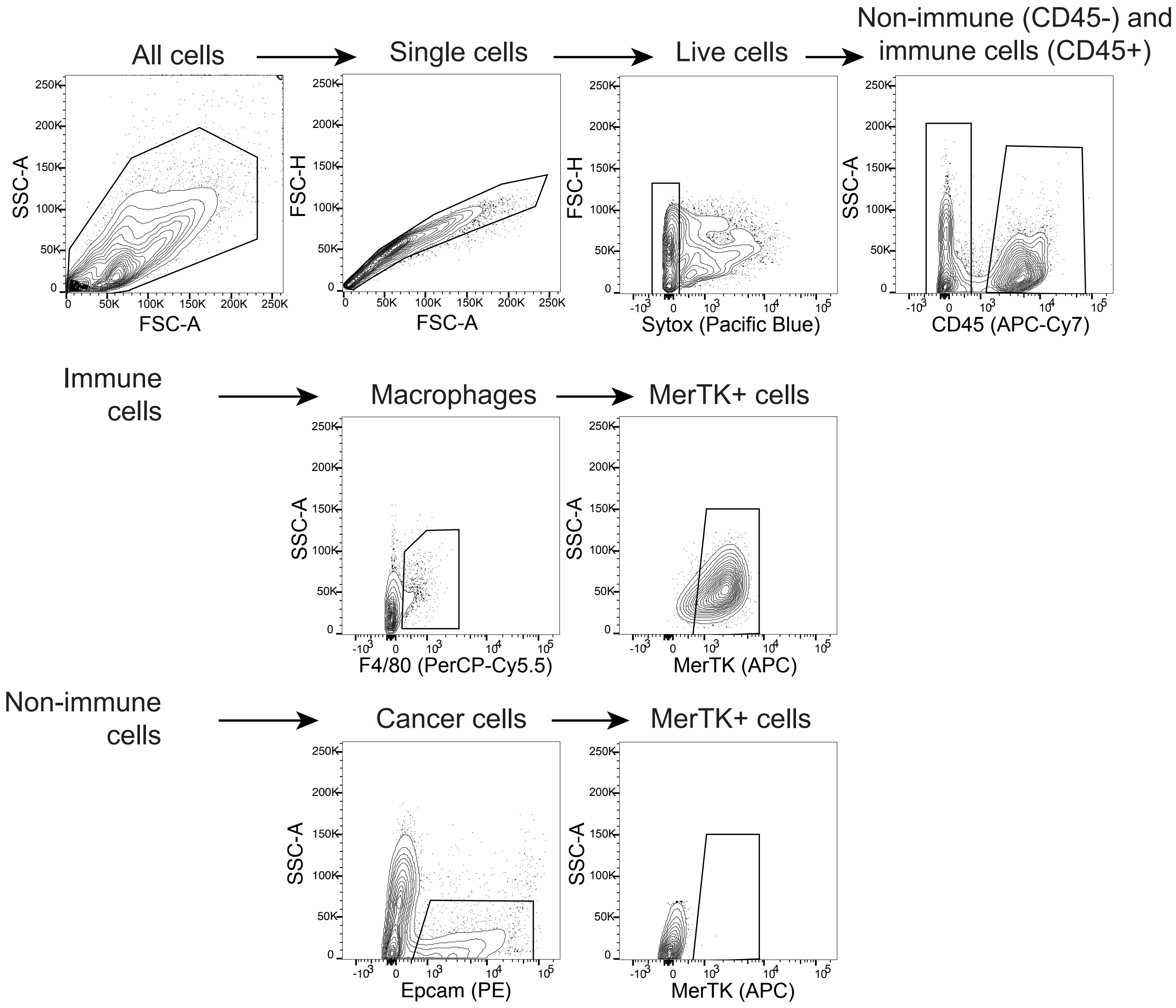

Supplementary Figure S6. FACS gating strategy of MerTK+ cells in spontaneous liver metastasis model using unlabelled KPC organoid.
