# Supplementary material for: Efferocytosis reprograms the tumor microenvironment to promote pancreatic cancer liver metastasis
Source: Nat Cancer. 2024 Feb 14;5(5):774–90. doi: 10.1038/s43018-024-00731-2 (PMC11136665; doi:10.1038/s43018-024-00731-2)
Supplement: Supplementary file 2 — Reporting Summary [file 43018_2024_731_MOESM2_ESM.pdf]

nature portfolio

Corresponding author(s): Michael C Schmid

Last updated by author(s): Dec 11, 2023

Reporting Summary

Nature Portfolio wishes to improve the reproducibility of the work that we publish. This form provides structure for consistency and transparency in reporting. For further information on Nature Portfolio policies, see our Editorial Policies and the Editorial Policy Checklist.

Statistics

For all statistical analyses, confirm that the following items are present in the figure legend, table legend, main text, or Methods section.

n/a	Confirmed	
☐	☒	The exact sample size (*n*) for each experimental group/condition, given as a discrete number and unit of measurement
☐	☒	A statement on whether measurements were taken from distinct samples or whether the same sample was measured repeatedly
☐	☒	The statistical test(s) used AND whether they are one- or two-sided<br>*Only common tests should be described solely by name; describe more complex techniques in the Methods section.*
☒	☐	A description of all covariates tested
☐	☒	A description of any assumptions or corrections, such as tests of normality and adjustment for multiple comparisons
☐	☒	A full description of the statistical parameters including central tendency (e.g. means) or other basic estimates (e.g. regression coefficient) AND variation (e.g. standard deviation) or associated estimates of uncertainty (e.g. confidence intervals)
☐	☒	For null hypothesis testing, the test statistic (e.g. F, t, r) with confidence intervals, effect sizes, degrees of freedom and P value noted<br>*Give P values as exact values whenever suitable.*
☒	☐	For Bayesian analysis, information on the choice of priors and Markov chain Monte Carlo settings
☒	☐	For hierarchical and complex designs, identification of the appropriate level for tests and full reporting of outcomes
☒	☐	Estimates of effect sizes (e.g. Cohen's *d*, Pearson's *r*), indicating how they were calculated

Our web collection on statistics for biologists contains articles on many of the points above.

Software and code

Policy information about availability of computer code

Data collection	Bulk RNAseq was performed on Illumina NextSeq500<br>scRNAseq was performed on Illumina NovaSeq 6000<br>RT qPCR was run on AriaMx Real Time PCR system<br>Flow cytometry was run on FACSCanto II<br>Cell sorting was performed on FACSAria III<br>Fluorescence imaging of cells was performed on LSM 800 or 900 microscope<br>Brightfield or fluorescence imaging of tissues was performed on Axio Observer Z1 microscope<br>Imaging of western blot membrane was performed on Chemidoc system
Data analysis	Sequencing data were processed through CellRanger 3.0.2 and analysed in R 3.6.1 using the Seurat library. Signature enrichment analysis was performed using GSVA. All code used to analyse data in this study is available on GitHub at https://github.com/CBFLivUni/Astuti_et_al_2023<br>Gene ontology enrichment analysis was performed using g:Profiler<br>PCR data were analysed using AriaMx software<br>Flow cytometry data were collected using FACSDiva and analysed using FlowJo v10<br>Microscopy imaging data were analysed using Zen 3.8 or CellTracker<br>Statistical tests were performed using GraphPad Prism 8

For manuscripts utilizing custom algorithms or software that are central to the research but not yet described in published literature, software must be made available to editors and reviewers. We strongly encourage code deposition in a community repository (e.g. GitHub). See the Nature Portfolio guidelines for submitting code & software for further information.

Data

Policy information about availability of data

All manuscripts must include a data availability statement. This statement should provide the following information, where applicable:

- Accession codes, unique identifiers, or web links for publicly available datasets
- A description of any restrictions on data availability
- For clinical datasets or third party data, please ensure that the statement adheres to our policy

Mouse scRNAseq were submitted to the Gene Expression Omnibus (GEO) repository and can be accessed under GEO accession no. GSE215118. Human bulk RNAseq data are available from the authors upon reasonable request and subsequent Data Transfer Agreement to protect patients' privacy.

Research involving human participants, their data, or biological material

Policy information about studies with human participants or human data. See also policy information about sex, gender (identity/presentation), and sexual orientation and race, ethnicity and racism.

Reporting on sex and gender	This information has not been collected
Reporting on race, ethnicity, or other socially relevant groupings	This information has not been collected
Population characteristics	Liver biopsy samples were collected from patients with treatment-naïve, advanced PDAC with pathologically confirmed liver metastasis. Blood samples were collected from patients with any stage of PDAC. Patients were 53-64 years old however age was not a consideration for sample collection.
Recruitment	Liver biopsy were part of diagnostic samples from consented patients with treatment-naïve, advanced PDAC at the Royal Liverpool University Hospital. Blood samples were collected from consented patients with any stage of PDAC. No biases were present.
Ethics oversight	Human studies were approved by the National Research Ethics Service (NRES). All individuals provided written informed consents for tissue donation on approved institutional protocol.

Note that full information on the approval of the study protocol must also be provided in the manuscript.

Field-specific reporting

Please select the one below that is the best fit for your research. If you are not sure, read the appropriate sections before making your selection.

☒ Life sciences ☐ Behavioural & social sciences ☐ Ecological, evolutionary & environmental sciences

For a reference copy of the document with all sections, see nature.com/documents/nr-reporting-summary-flat.pdf

Life sciences study design

All studies must disclose on these points even when the disclosure is negative.

Sample size	No statistical methods were used to pre-determine sample sizes but we used adequate numbers of samples that would provide statistically significant results based on our previous experience with the tumour models. At least 3 mice were analysed for each group in the animal studies.
Data exclusions	No data were excluded from the analyses.
Replication	Biological replicates are defined for each experiment in the figure legends. Animal studies were performed twice, with the exceptions of the MerTKi and Grn KO spontaneous liver metastasis studies, which were performed once. In vitro experiments were repeated three times unless otherwise specified in the figure legends.
Randomization	The experiments were not randomised, however mice with comparable age and body weight were assigned into control and experimental groups. The exception of this was the MerTKi study in spontaneous liver metastasis model where, prior to drug treatment, mice with comparable primary tumour burden were randomly assigned.
Blinding	The Investigators were not blinded to allocation during experiments and outcome assessment. Treatments, data collections, and analyses were performed by the same person so blinding was not possible. In some instances, imaging data were acquired by a different person who did not have knowledge of expected treatment outcome.

Behavioural & social sciences study design

All studies must disclose on these points even when the disclosure is negative.

Study description	N/A
Research sample	N/A
Sampling strategy	N/A
Data collection	N/A
Timing	N/A
Data exclusions	N/A
Non-participation	N/A
Randomization	N/A

Ecological, evolutionary & environmental sciences study design

All studies must disclose on these points even when the disclosure is negative.

Study description	N/A
Research sample	N/A
Sampling strategy	N/A
Data collection	N/A
Timing and spatial scale	N/A
Data exclusions	N/A
Reproducibility	N/A
Randomization	N/A
Blinding	N/A

Did the study involve field work? ☐ Yes ☒ No

Reporting for specific materials, systems and methods

We require information from authors about some types of materials, experimental systems and methods used in many studies. Here, indicate whether each material, system or method listed is relevant to your study. If you are not sure if a list item applies to your research, read the appropriate section before selecting a response.

Materials & experimental systems

n/a	Involved in the study	
☐	☒	Antibodies
☐	☒	Eukaryotic cell lines
☒	☐	Palaeontology and archaeology
☐	☒	Animals and other organisms
☒	☐	Clinical data
☒	☐	Dual use research of concern
☒	☐	Plants

Methods

n/a	Involved in the study	
☒	☐	ChIP-seq
☐	☒	Flow cytometry
☒	☐	MRI-based neuroimaging

Antibodies

Antibodies used

Primary Antibodies
Antigen - Clone - Manufacturer - Catalogue number - Application - Dilution
Rat anti-CD8 53-6.7 Biolegend 100734 FACS 1:100
Rat anti-IFNg XMG1.2 Biolegend 505808 FACS 1:100
Rat anti-CD11b M1/70 Biolegend 101216 FACS 1:100
Rat anti-TIM4 RMT4-54 Biolegend 130005 FACS 1:100
Hamster anti-CD69 H1.2F3 Biolegend 104507 FACS 1:100
Rat anti-CD45 30-F11 Biolegend 103116 FACS 1:100
Rat anti-granzyme B GB11 Biolegend 515405 FACS 1:100
Rat anti-F4/80 BM8 Biolegend 123115/123128 FACS 1:100
Rat anti-CD74 In1/CD74 Biolegend 151004 FACS 1:100
Rat anti MerTK 2B10C42 Biolegend 151508 FACS 1:100
Rabbit anti-CK19 (for human tissues) Polyclonal Abcam ab53119 IF 1:500
Rabbit anti-CK19 (for mouse tissues) EPNCIR127B Abcam ab133496 IF 1:500
Mouse anti-CD68 KP1 Dako M081401-2 IF 1:100
Rabbit anti-F4/80 D2S9R Cell Signaling Technologies 70076 IF 1:100
Rabbit anti-VSIG4 EPR22576-70 Abcam ab252933 IF 1:100
Rat anti-CD8 (for human tissues) 144b Dako M710301-2 IF 1:100
Rat anti-CD8 (for mouse tissues) 53-6.7 eBioscience (Thermo Fisher) 14-0081-82 IF 1:100
Rat anti-LAMP1 1D4B Abcam ab25245 IF 1:200
Rabbit anti-granzyme B (for mouse tissues) Polyclonal Abcam ab4059 IF 1:100
Mouse anti-granzyme B (for human tissues) GrB-7 Dako M7235 IF 1:100
Rat anti-F4/80 BM8 Biolegend 123102 IF 1:100
Rabbit anti-F4/80 D2S9R Cell Signaling Technologies 70076 IF 1:100
Rat anti-CD74 In1/CD74 Biolegend 151002 IF 1:100
Mouse anti-CFTR A-3 Santa Cruz sc376683 IF/PLA 1:50
Rabbit anti-mCherry Polyclonal Abcam ab167453 IF/PLA 1:500
Rabbit anti-YM-1 Polyclonal Stem Cell Technologies 60130 IF 1:100
Mouse anti-LXR? PPZ0412 Abcam ab41902 PLA 1:100
Rabbit anti-RXR? EPR7106 Abcam ab125001 PLA 1:100
Rabbit anti-IRF3 D83B9 Cell Signaling Technologies 4302 WB 1:1000
Rabbit anti-Histone H3 Polyclonal Abcam ab1791 WB 1:10,000
Rabbit anti-Arginase 1 Polyclonal Proteintech 16001-1-AP WB 1:1000
Rabbit anti-Cofilin D3F9 Cell Signaling Technologies 5175 WB 1:10,000

Secondary Antibodies
Name - Clone - Manufacturer - Catalogue number - Application - Dilution
Alexa Fluor® 488-donkey anti-mouse Polyclonal Abcam ab150105 IF 1:500
Alexa Fluor® 594-donkey anti-rabbit Polyclonal Biolegend 406418 IF 1:500
Alexa Fluor® 647-donkey anti-rat Polyclonal Abcam ab150155 IF 1:500
DyLight 488-goat anti-rat Polyclonal Abcam ab96887 IF 1:500
Alexa Fluor® 647-goat anti-rabbit Polyclonal Abcam ab150079 IF 1:500
Alexa Fluor® 488-goat anti-rabbit Polyclonal Abcam ab150077 IF 1:500
Alexa Fluor® 647-goat anti-rat Polyclonal Abcam ab150159 IF 1:500
Alexa Fluor® 594-goat anti rat Polyclonal Abcam ab150160 IF 1:500
HRP-goat anti-rabbit Polyclonal Cell Signaling Technologies 7074 WB 1:5000

Validation

All antibodies have been validated by the manufacturers, as indicated in the references provided.

Primary antibodies
Rat anti-CD8 53-6.7 Biolegend 100734 https://www.biolegend.com/en-gb/search-results/percp-cyanine5-5-anti-mouse-cd8a-antibody-4255
Rat anti-IFNg XMG1.2 Biolegend 505808 https://www.biolegend.com/en-gb/antibodies-and-more/pe-anti-mouse-ifn-gamma-antibody-997?GroupID=GROUP24
Rat anti-CD11b M1/70 Biolegend 101216 https://www.biolegend.com/en-gb/products/pe-cyanine7-anti-mouse-human-cd11b-antibody-1921
Rat anti-TIM4 RMT4-54 Biolegend 130005 https://www.biolegend.com/en-gb/products/pe-anti-mouse-tim-4-antibody-5242
Hamster anti-CD69 H1.2F3 Biolegend 104507 https://www.biolegend.com/en-gb/products/pe-anti-mouse-cd69-antibody-265
Rat anti-CD45 30-F11 Biolegend 103116 https://www.biolegend.com/en-gb/products/apc-cyanine7-anti-mouse-cd45-antibody-2530
Rat anti-granzyme B GB11 Biolegend 515405 https://www.biolegend.com/en-gb/products/alexa-fluor-647-anti-human-mouse-granzyme-b-antibody-6067
Rat anti-F4/80 BM8 Biolegend 123115 https://www.biolegend.com/en-gb/products/apc-anti-mouse-f4-80-antibody-4071
Rat anti-F4/80 BM8 Biolegend 123128 https://www.biolegend.com/en-gb/products/percp-cyanine5-5-anti-mouse-f480-antibody-4303
Rat anti-CD74 In1/CD74 Biolegend 151004 https://www.biolegend.com/en-gb/products/alexa-fluor-647-anti-mouse-cd74-clip-antibody-13701
Rat anti MerTK 2B10C42 Biolegend 151508 https://www.biolegend.com/en-gb/products/apc-anti-mouse-mertk-mer-antibody-13980
Rabbit anti-CK19 (for human tissues) Polyclonal Abcam ab53119 https://www.abcam.com/products/primary-antibodies/cytokeratin-19-antibody-ab53119.html
Rabbit anti-CK19 (for mouse tissues) EPNCIR127B Abcam ab133496 https://www.abcam.com/products/primary-antibodies/cytokeratin-19-antibody-epncir127b-ab133496.html
Mouse anti-CD68 KP1 Dako M081401-2

Rabbit anti-F4/80 D2S9R Cell Signaling Technologies 70076 https://www.cellsignal.com/products/primary-antibodies/f4-80-d2s9r-xp-rabbit-mab/70076
Rabbit anti-VSIG4 EPR22576-70 Abcam ab252933 https://www.abcam.com/products/primary-antibodies/vsig4-antibody-epr22576-70-ab252933.html
Rat anti-CD8 (for human tissues) CD8/144b Dako M710301-2 https://www.agilent.com/en/product/immunohistochemistry/antibodies-controls/primary-antibodies/cd8-%28concentrate%29-76631
Rat anti-CD8 (for mouse tissues) 53-6.7 eBioscience (Thermo Fisher) 14-0081-82 https://www.thermofisher.com/antibody/product/CD8a-Antibody-clone-53-6-7-Monoclonal/14-0081-82
Rat anti-LAMP1 1D4B Abcam ab25245 https://www.abcam.com/products/primary-antibodies/lamp1-antibody-1d4b-ab25245.html
Rabbit anti-granzyme B (for mouse tissues) Polyclonal Abcam ab4059 https://www.abcam.com/products/primary-antibodies/granzyme-b-antibody-ab4059.html
Mouse anti-granzyme B (for human tissues) GrB-7 Dako M7235 https://www.agilent.com/en/product/immunohistochemistry/antibodies-controls/primary-antibodies/granzyme-b-%28concentrate%29-76643
F4/80 BM8 Biolegend 123102 https://www.biolegend.com/en-gb/products/purified-anti-mouse-f4-80-antibody-4064
Rat anti-CD74 In1/CD74 Biolegend 151002 https://www.biolegend.com/en-gb/products/purified-anti-mouse-cd74-clip-antibody-12565
Mouse anti-CFTR A-3 Santa Cruz sc376683 https://www.scbt.com/p/cftr-antibody-a-3
Rabbit anti-mCherry Polyclonal Abcam ab167453 https://www.abcam.com/products/primary-antibodies/mcherry-antibody-ab167453.html
Rabbit anti-YM-1 Polyclonal Stem Cell Technologies 60130 https://www.stemcell.com/products/anti-ym1-antibody-polyclonal.html
Mouse anti-LXR? PPZ0412 Abcam ab41902 https://www.abcam.com/products/primary-antibodies/lxr-alpha-antibody-ppz0412-ab41902.html
Rabbit anti-RXR? EPR7106 Abcam ab125001 https://www.abcam.com/products/primary-antibodies/retinoid-x-receptor-alpharxra-antibody-epr7106-ab125001.html
Rabbit anti-IRF3 D83B9 Cell Signaling Technologies 4302
Rabbit anti-Histone H3 Polyclonal Abcam ab1791 https://www.abcam.com/products/primary-antibodies/histone-h3-antibody-nuclear-marker-and-chip-grade-ab1791.html
Rabbit anti-Arginase 1 Polyclonal Proteintech 16001-1-AP https://www.ptglab.com/products/ARG1-Antibody-16001-1-AP.htm
Rabbit anti-Cofilin D3F9 Cell Signaling Technologies 5175 https://www.cellsignal.com/products/primary-antibodies/cofilin-d3f9-xp-rabbit-mab/5175

Secondary antibodies
Alexa Fluor® 488-donkey anti-mouse Polyclonal Abcam ab150105 https://www.abcam.com/products/secondary-antibodies/donkey-mouse-igg-hl-alexa-fluor-488-ab150105.html
Alexa Fluor® 594-donkey anti-rabbit Polyclonal Biolegend 406418
DyLight 488-goat anti-rat Polyclonal Abcam ab96887 https://www.abcam.com/products/secondary-antibodies/goat-rat-igg-hl-dylight-488-ab96887.html
Alexa Fluor® 647-goat anti-rabbit Polyclonal Abcam ab150079 https://www.abcam.com/products/secondary-antibodies/goat-rabbit-igg-hl-alexa-fluor-647-ab150079.html
Alexa Fluor® 488-goat anti-rabbit Polyclonal Abcam ab150077 https://www.abcam.com/products/secondary-antibodies/goat-rabbit-igg-hl-alexa-fluor-488-ab150077.html
Alexa Fluor® 647-goat anti-rat Polyclonal Abcam ab150159 https://www.abcam.com/products/secondary-antibodies/goat-rat-igg-hl-alexa-fluor-647-ab150159.html
Alexa Fluor® 594-goat anti rat Polyclonal Abcam ab150160 https://www.abcam.com/products/secondary-antibodies/goat-rat-igg-hl-alexa-fluor-594-ab150160.html
Alexa Fluor® 647-donkey anti-rat Polyclonal Abcam ab150155 https://www.abcam.com/products/secondary-antibodies/donkey-rat-igg-hl-alexa-fluor-647-preadsorbed-ab150155.html
HRP-goat anti-rabbit Polyclonal Cell Signaling Technologies 7074 https://www.cellsignal.com/products/secondary-antibodies/anti-rabbit-igg-hrp-linked-antibody/7074

Eukaryotic cell lines

Policy information about cell lines and Sex and Gender in Research

Cell line source(s)	PDAC cells derived from KPC mice on C57BL/6 background were provided by Dr. David Tuveson at the Cold Spring Harbor Laboratory. Liver metastatic organoid cultures from KPC mice on C57BL/6 background were created by Michael Schmid lab, University of Liverpool, UK. HEK293T, THP-1, and Jurkat cells were obtained from ATCC.
Authentication	KPC cells were authenticated by genotyping PCR. HEK293T, THP-1, and Jurkat cells were authenticated by the suppliers.
Mycoplasma contamination	All cell lines were routinely tested negative for mycoplasma.
Commonly misidentified lines (See ICLAC register)	No commonly misidentified cell lines were used in this study.

Animals and other research organisms

Policy information about studies involving animals; ARRIVE guidelines recommended for reporting animal research, and Sex and Gender in Research

Laboratory animals	C57BL/6 mice were obtained from Charles River Laboratories. Grn-/- (B6(Cg)-Grntm1.1Aidi), Grnfl/fl (C57BL/6-Grntm1Aidi), and tdTomato+ mice (B6.129(Cg)–Gt(ROSA)26Sortm4(ACTB–tdTomato,–EGFP)Luo) on the C57BL/6 genetic background were purchased from the Jackson Laboratory. Tamoxifen inducible Csf1r-Cre mice (Csf1r-Mer-iCre-Mer) on the C57BL/6 background were provided by

	Dr. Jeffrey W. Pollard at the University of Edinburgh.
Wild animals	No wild animals were used in the animal studies.
Reporting on sex	Female mice were used, except for the spontaneous liver metastasis study, which used male mice.
Field-collected samples	No field-collected samples were used in this study.
Ethics oversight	All animal procedures were conducted in accordance with the UK Home Office regulations under the project licence P16F36770. Maximum tumour burden limit of 1.5 cm mean diameter was not exceeded in the studies. In all animal studies, severity was limited to moderate.

Note that full information on the approval of the study protocol must also be provided in the manuscript.

Plants

Seed stocks	N/A
Novel plant genotypes	N/A
Authentication	N/A

Flow Cytometry

Plots

Confirm that:

☒ The axis labels state the marker and fluorochrome used (e.g. CD4-FITC).

☒ The axis scales are clearly visible. Include numbers along axes only for bottom left plot of group (a 'group' is an analysis of identical markers).

☒ All plots are contour plots with outliers or pseudocolor plots.

☒ A numerical value for number of cells or percentage (with statistics) is provided.

Methodology

Sample preparation	Single-cell suspensions from murine livers were prepared by mechanical and enzymatic disruption with 1 mg/mL Collagenase P (Roche) in Hanks Balanced Salt Solution (HBSS) at 37C for 30-40 minutes. Cells were then incubated with 0.05% trypsin at 37C for 5 minutes. After removal of debris by filtering the cell suspension through a 70um strainer, red blood cells were removed using RBC Lysis Buffer (Biolegend).<br>Liver cell suspensions were then resuspended in MACS buffer (0.5% BSA, 2mM EDTA, PBS). Fc receptors were blocked using anti-mouse CD16/CD32 (BD Biosciences) for 10 minutes on ice. For cell surface staining, cells were then incubated with SYTOX Blue viability marker (Thermo Fisher) and fluorophore-conjugated antibodies (Biolegend, Supplementary Table 12).<br>For the T cell activation assay, following Fc receptor blocking, cells were incubated LIVE/DEAD™ Fixable Aqua Dead Cell Stain Kit (Thermo Fisher) and fluorophore-conjugated CD8 antibody (Biolegend). Cells were then fixed using IC Fixation Buffer and permeabilised using Intracellular Staining Perm Wash Buffer (Biolegend) according to the manufacturer's instructions, followed by staining with fluorophore-conjugated IFNg antibody.<br>For T cell activation assay, following Fc receptor blocking, cells were incubated LIVE/DEAD™ Fixable Aqua Dead Cell Stain Kit (Thermo Fisher) and fluorophore-conjugated CD8 antibody (Biolegend). Cells were then fixed using IC Fixation Buffer and permeabilised using Intracellular Staining Perm Wash Buffer (Biolegend) according to the manufacturer's instructions, followed by staining with fluorophore-conjugated IFNγ and granzyme B antibodies (Biolegend, Supplementary Table 4).
Instrument	FACSCanto II<br>FACSAria III (for cell sorting)
Software	BD FACSDiva software
Cell population abundance	FACS-sorted cell abundance and purity was determined using FACSDiva software
Gating strategy	Single cells were gated based on FSC-A and FSC-H. Live cells were gated based on Sytox or LIVE/DEAD staining. Macrophages were gated based on CD45+ F4/80+ staining. CD8 T cells from in vivo samples were gated based on CD45+ CD3+ CD8+ staining. CD8 T cells from in vitro samples were gated based on CD8+.

☒ Tick this box to confirm that a figure exemplifying the gating strategy is provided in the Supplementary Information.
