# Supplementary figures and images for: Efferocytosis reprograms the tumor microenvironment to promote pancreatic cancer liver metastasis
Source: Nat Cancer. 2024 Feb 14;5(5):774–90. doi: 10.1038/s43018-024-00731-2 (PMC11136665; doi:10.1038/s43018-024-00731-2)

FIGURE 3h

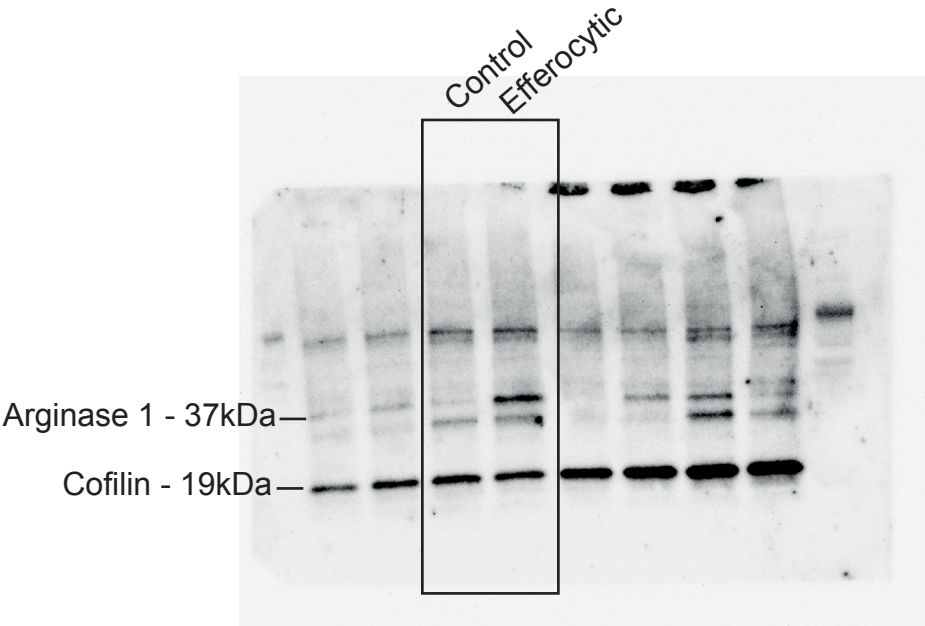

**FIGURE 5e**

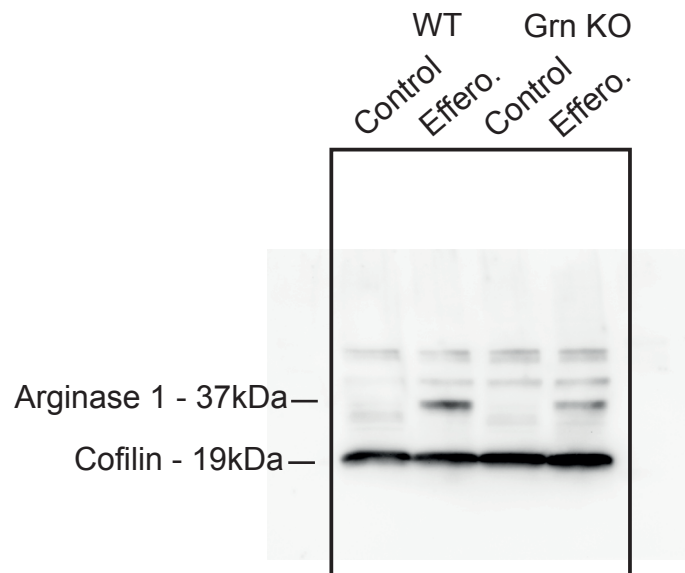

**FIGURE 7c**

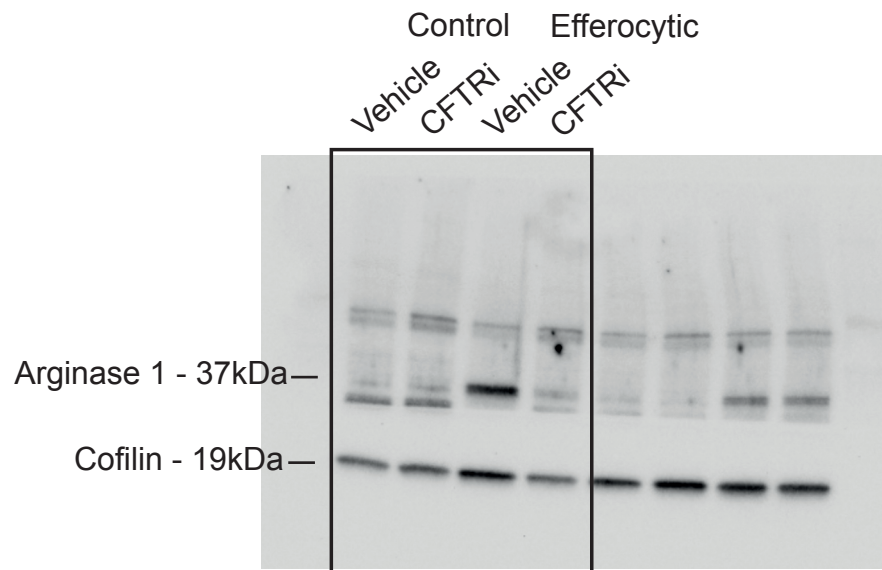

EXTENDED DATA FIGURE 4a

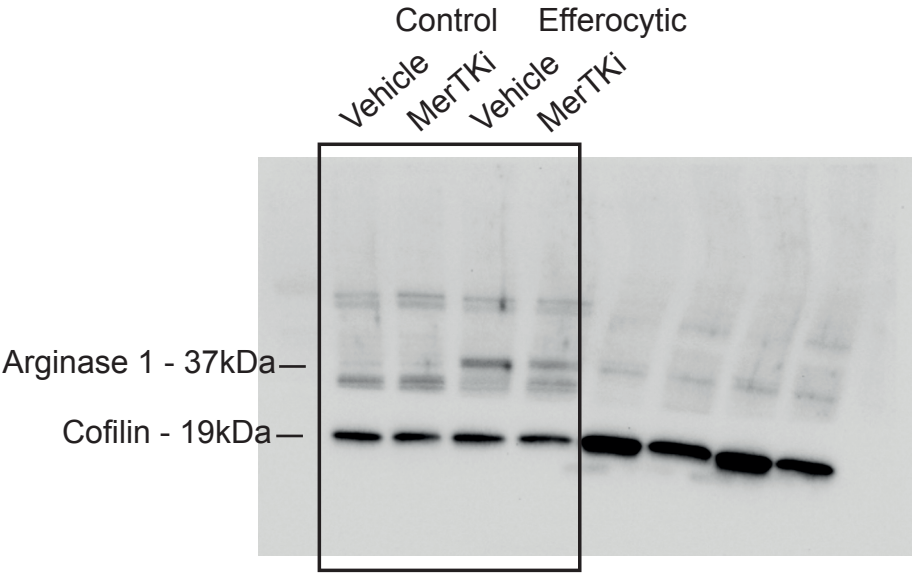

EXTENDED DATA FIGURE 7f

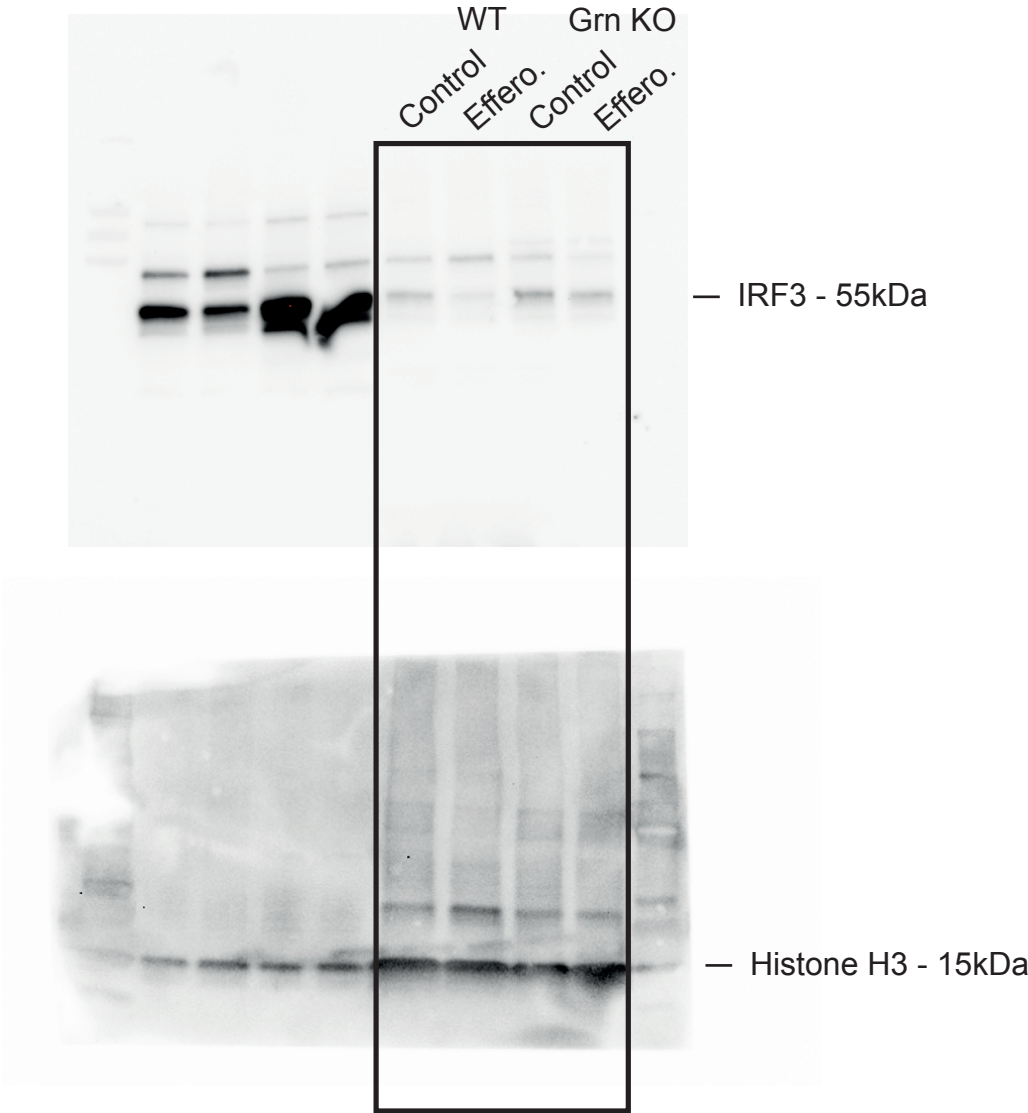

Supplement: Supplementary file 19 — Unprocessed western blot images. [file 43018_2024_731_MOESM19_ESM.pdf]
